# Connective Auxin Transport in the Shoot Facilitates Communication between Shoot Apices

**DOI:** 10.1371/journal.pbio.1002446

**Published:** 2016-04-27

**Authors:** Tom Bennett, Geneviève Hines, Martin van Rongen, Tanya Waldie, Megan G. Sawchuk, Enrico Scarpella, Karin Ljung, Ottoline Leyser

**Affiliations:** 1 Sainsbury Laboratory, University of Cambridge, Bateman Street, Cambridge, United Kingdom; 2 Department of Biological Sciences, University of Alberta, Edmonton, Alberta, Canada; 3 Umeå Plant Science Centre, Department of Forest Genetics and Plant Physiology, Swedish University of Agricultural Sciences, Umeå, Sweden; University of North Carolina, UNITED STATES

## Abstract

The bulk polar movement of the plant signaling molecule auxin through the stem is a long-recognized but poorly understood phenomenon. Here we show that the highly polar, high conductance polar auxin transport stream (PATS) is only part of a multimodal auxin transport network in the stem. The dynamics of auxin movement through stems are inconsistent with a single polar transport regime and instead suggest widespread low conductance, less polar auxin transport in the stem, which we term connective auxin transport (CAT). The bidirectional movement of auxin between the PATS and the surrounding tissues, mediated by CAT, can explain the complex auxin transport kinetics we observe. We show that the auxin efflux carriers PIN3, PIN4, and PIN7 are major contributors to this auxin transport connectivity and that their activity is important for communication between shoot apices in the regulation of shoot branching. We propose that the PATS provides a long-range, consolidated stream of information throughout the plant, while CAT acts locally, allowing tissues to modulate and be modulated by information in the PATS.

## Abstract 2

The growing shoot tips of a plant can communicate and influence each other’s growth. This study reveals that this communication is mediated by a multimodal auxin transport network in the stem, with complex dynamics.

## Author Summary

The growing shoot tips of a plant can communicate and influence each other’s growth. This is most obvious in the phenomenon of apical dominance, in which the leading shoot in a shoot system inhibits the growth of buds in the axils of the leaves below it. Removing the leading shoot results in the activation of these buds to produce branches. It has been known for almost a century that the plant hormone auxin plays a central part in apical dominance. Auxin is produced in growing shoot tips and transported down the main stem in the Polar Auxin Transport Stream (PATS). We have previously shown that communication between shoot tips is mediated at least in part by competition between buds for access to the PATS. Here we show that this communication depends not only on the highly polar, high-capacity transport of the PATS, which is localized to the vascular bundles of the stem, but also on widespread less polar transport activities, which act to exchange information between the PATS and surrounding tissues. We term this Connective Auxin Transport (CAT) and show that the exchange of auxin between CAT and PATS is important for bud-bud communication.

## Introduction

The polar auxin transport stream (PATS) can be defined as a long distance transport route carrying the plant hormone auxin from growing shoot tips towards the roots. The phenomenon of polar auxin transport was integral to the discovery of auxin, providing the first experimental source of the “growth substance,” later shown to be auxin, which was collected from exudates of young coleoptiles or shoot tips [1,2]. The PATS can be observed by tracking the movement of exogenously applied radio-labelled auxin through the stems of many plant species (reviewed in [3]). The polar nature of auxin transport led to the development of the chemiosmotic theory of auxin transport, in which protonated indole-3-acetic acid (IAA) molecules in the apoplast (~pH 5.5) can enter cells across the plasma membrane, but the largely deprotonated population of IAA molecules in the cytoplasm (~pH 7) cannot efficiently exit the cell without efflux carriers, which can be polarly localized [4,5]. Twenty years later, molecular genetic studies in *Arabidopsis thaliana* identified such a family of auxin efflux carriers—the PIN-FORMED (PIN) proteins—which are often polarly localized [6–9]. This family includes the PIN1 protein, which has been shown to be polarly localized in the xylem parenchyma and vascular cambium of stems, which are major sites for the PATS [3,9,10]. An important role for PIN1 in the PATS is further supported by the observation that in *pin1* mutants polar auxin transport in stems is significantly reduced [11,12].

Further analysis of PIN proteins has demonstrated their central contributions to other auxin-regulated processes, particularly in patterning events (reviewed in [13]). For example, in the shoot apex, the dynamic re-localization of PIN1 is involved in phyllotaxis [14] and leaf vein patterning (reviewed in [15]). The involvement of PIN proteins in patterning is likely to be conserved across the land plants, and as such, PIN protein-mediated auxin redistribution can be considered a fundamental patterning mechanism in plants [16–18]. These patterning processes involve local fluxes of auxin across relatively short-distances, in contrast to the long-range mobilization of auxin observed in the PATS. However, much of the auxin involved in local patterning at the shoot apex and in developing leaves is loaded into the PATS and transported rootward. Local patterning events and the PATS are thus interconnected.

Three major roles for the PATS have been suggested. Firstly, auxin in the PATS promotes cambial activity leading to the secondary growth of stems [19]. This function is consistent with the observation that cambium is a major site of the PATS [20]. Secondly, since auxin moving in the PATS provides information about the activity of the shoot apices that feed auxin into it and is ultimately delivered to the root, PATS provides a mechanism for shoot-root communication. For example, shoot-derived auxin has been shown to influence development in the roots of young Arabidopsis seedlings [21,22]. Thirdly, it has been proposed that the PATS plays a central role in the dynamic control of shoot architecture. Shoot systems in flowering plants are built through the action of shoot apical meristems (SAMs), which produce leaves at their periphery and stem tissue underneath. In the axil of each leaf, a new axillary SAM is established. These axillary meristems can either remain dormant as a bud or activate to produce a branch. One of the earliest identified roles for the PATS was in the inhibition of axillary buds by auxin transported rootward from the shoot tip. Removal of the shoot tip, i.e., decapitation, results in the activation of axillary buds, but application of auxin to the decapitation site can restore inhibition [23], though not if auxin transport inhibitors are simultaneously applied [24]. Auxin from the shoot tip does not enter the bud in appreciable amounts and therefore acts indirectly to regulate shoot branching [25–27].

One proposed mechanism by which auxin in the PATS could inhibit buds indirectly is by regulating the synthesis of second messengers that can move into buds to modulate their activity. Consistent with this idea, auxin in the stem up-regulates the expression of biosynthesis genes for strigolactones, which can inhibit bud activation [28], and down-regulates the expression of genes involved in the biosynthesis of cytokinins, which can promote bud activity [29]. However, these effects are insufficient to explain a range of observations regarding shoot branching control by auxin in the PATS. For example, in Arabidopsis, decapitation still releases the buds of cytokinin deficient or resistant mutants from apical dominance [29], and treatment with strigolactone can actually promote the activation of buds in mutants with compromised auxin transport [12].

An alternative, non-exclusive explanation is that the dynamic properties of the auxin transport system are central in mediating the indirect effect of auxin on bud growth inhibition. This model is based on Sachs’ auxin transport canalization hypothesis, in which an initially passive flux of auxin from a source to a sink up-regulates and polarizes auxin transport in the direction of the flux, such that a broad domain of auxin transport becomes a narrow, highly polarized “canal” that determines the position of vascular strand differentiation [30,31]. A modern, PIN-based re-statement of the canalization hypothesis thus supposes that in response to local auxin application, auxin flux from this source to the nearest sink should drive up-regulation of PIN proteins in the intermediate tissues coupled with increased PIN polarization in the direction of the sink, resulting in gradual narrowing of the domain of PIN expression. An emerging model for shoot branching control is that by acting as sink for auxin, the PATS allows canalization-driven auxin export from axillary buds, which are auxin sources, and this is hypothesized to be required for bud outgrowth [10,12,26,32–35]. According to this model, lateral buds effectively compete for access to the PATS in the main stem. The stronger the sink provided by the PATS, the more buds are able to activate [33]. It is not only the concentration of auxin in the PATS that is important, but also its ability to transport auxin away towards the root, since both factors contribute to sink strength. Thus, in this context, the PATS functions to facilitate communication between apices in a shoot system.

We have previously analyzed this hypothesis using metamer-level and pseudo-cellular scale mathematical modelling of auxin transport, and shown that the generalized properties of canalization-dependent auxin transport can give rise to observed patterns of bud activation [33]. However these models represent the PATS in a highly simplified manner and cannot easily account for some basic attributes of the system such as the fact that buds on opposite sides of the stem can inhibit each other’s activity despite exporting their auxin into separate vascular bundles [33,36].

In order to understand better the role of the PATS in shoot branching control, we have undertaken an in-depth re-assessment of the properties of auxin transport and the behavior of PIN proteins in Arabidopsis inflorescence stems. We provide evidence that the movement of auxin in stems is not consistent with a transport system dominated by a single highly polarized regime, but rather is better modelled by a scenario with two or three different cellular auxin transport regimes, with exchange of auxin between them. Analysis of auxin transporters in the stem supports our model and suggests that these different regimes are tissue- rather than PIN-specific. PIN1 is the major contributor to a classical highly polar, high-conductance PATS, while PIN3, PIN4, and PIN7 contribute to a broader low-conductance and less polar activity, acting at the interface between the PATS and broader stem tissues. We conclude that auxin transport in the stem can be considered as a combination of a highly stable PATS, which integrates information across the shoot system and between the shoot and the root, and transport activities that we term Connective Auxin Transport (CAT), which mediate two-way communication between the PATS and surrounding tissues, including axillary meristems.

## Results

### PIN1 Responds Slowly to Stem Decapitation

**Fig 1:**
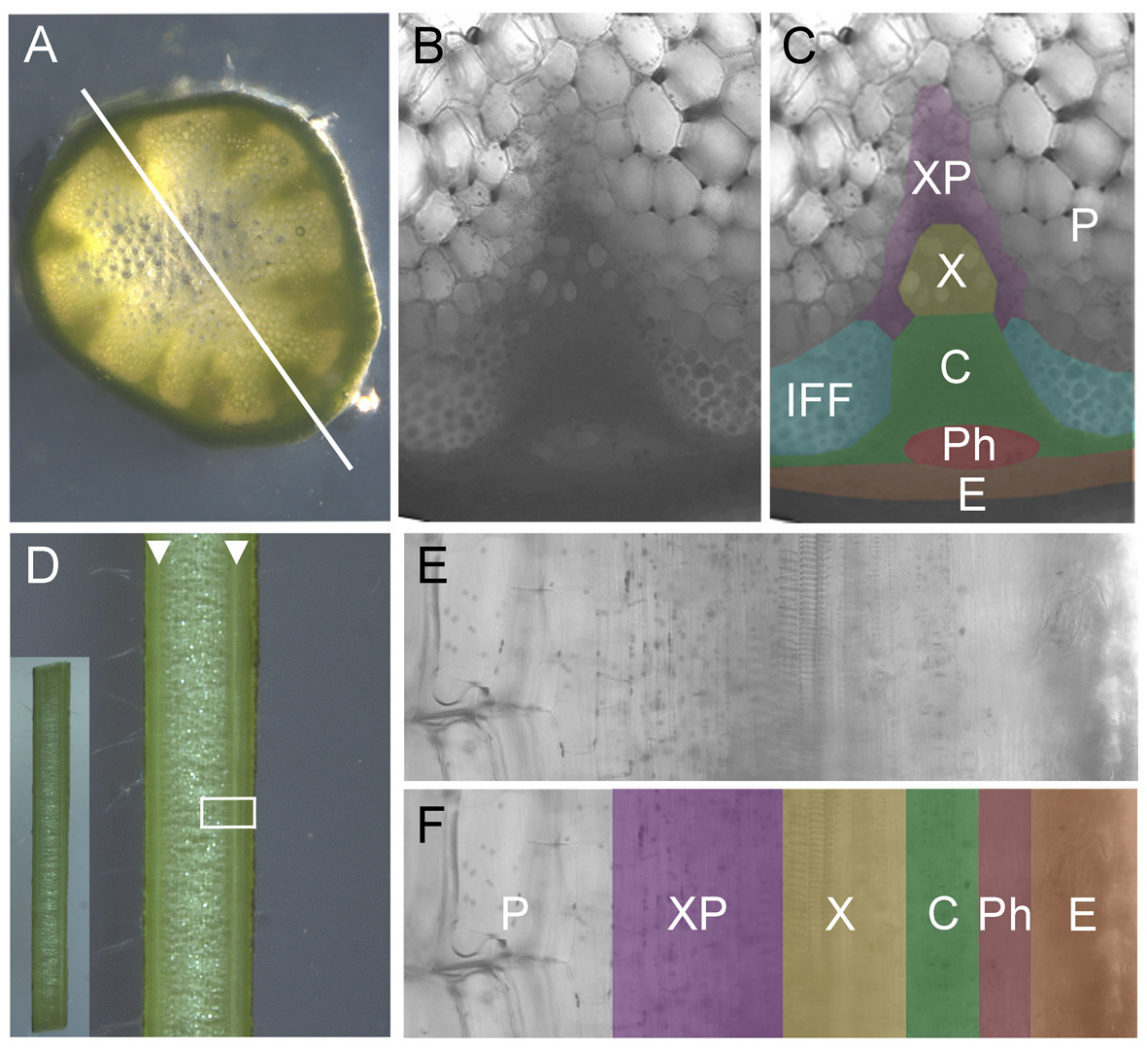
Anatomy of Arabidopsis inflorescence stems. **A)** Light micrograph of transverse section through the basal internode of a 6-wk-old Arabidopsis inflorescence stem. Vascular bundles are clearly visible as dark green triangles. Longitudinal sections used in this study were made across the center of the stem, between two vascular bundles, as indicated by the white line. **B, C)** Close up of cellular anatomy in a transverse section of an Arabidopsis vascular bundle and surrounding tissue. (C) is shaded to indicate tissue types. **D)** Light micrograph of longitudinal section through the basal internode of a 6-wk-old Arabidopsis inflorescence stem (along the type of transect indicated in A). Vascular bundles are visible as continuous white lines (indicated by white arrow heads). Inset shows the whole 2 cm segment. White box indicates the region shown in E, F. **E,F)** Close up of cellular anatomy in longitudinal section of an Arabidopsis vascular bundle and surrounding tissue. (F) is shaded to indicate tissue types. Unshaded/P = pith, purple/XP = xylem parenchyma, yellow/X = xylem, green/C = cambium, red/Ph = phloem, orange/E = epidermis, blue/IFF = interfascicular fibers.

In our previously reported basic model for shoot branching control the auxin transport network across the stem is considered as a unitary system, with the plasma membrane accumulation of a generic PIN protein established and maintained by a canalization-based mechanism [33]. If this assumption is correct, PIN accumulation and polarization in stems should respond strongly to auxin flux. To test this hypothesis, we used isolated 2cm stem segments from basal primary inflorescence internodes of 5- or 6-wk-old plants expressing a well-characterized PIN1-GFP fusion protein from its native promoter, which complements the *pin1* mutant [37]. This construct is expressed in the xylem parenchyma and cambial cells of the stem vascular bundles (stem anatomy is summarized in Fig 1), which are tissues classically associated with the PATS [20,38]. This expression pattern is identical to that of a different *PIN1pro*:*PIN1-GFP* line described previously [10,39].

**Fig 2:**
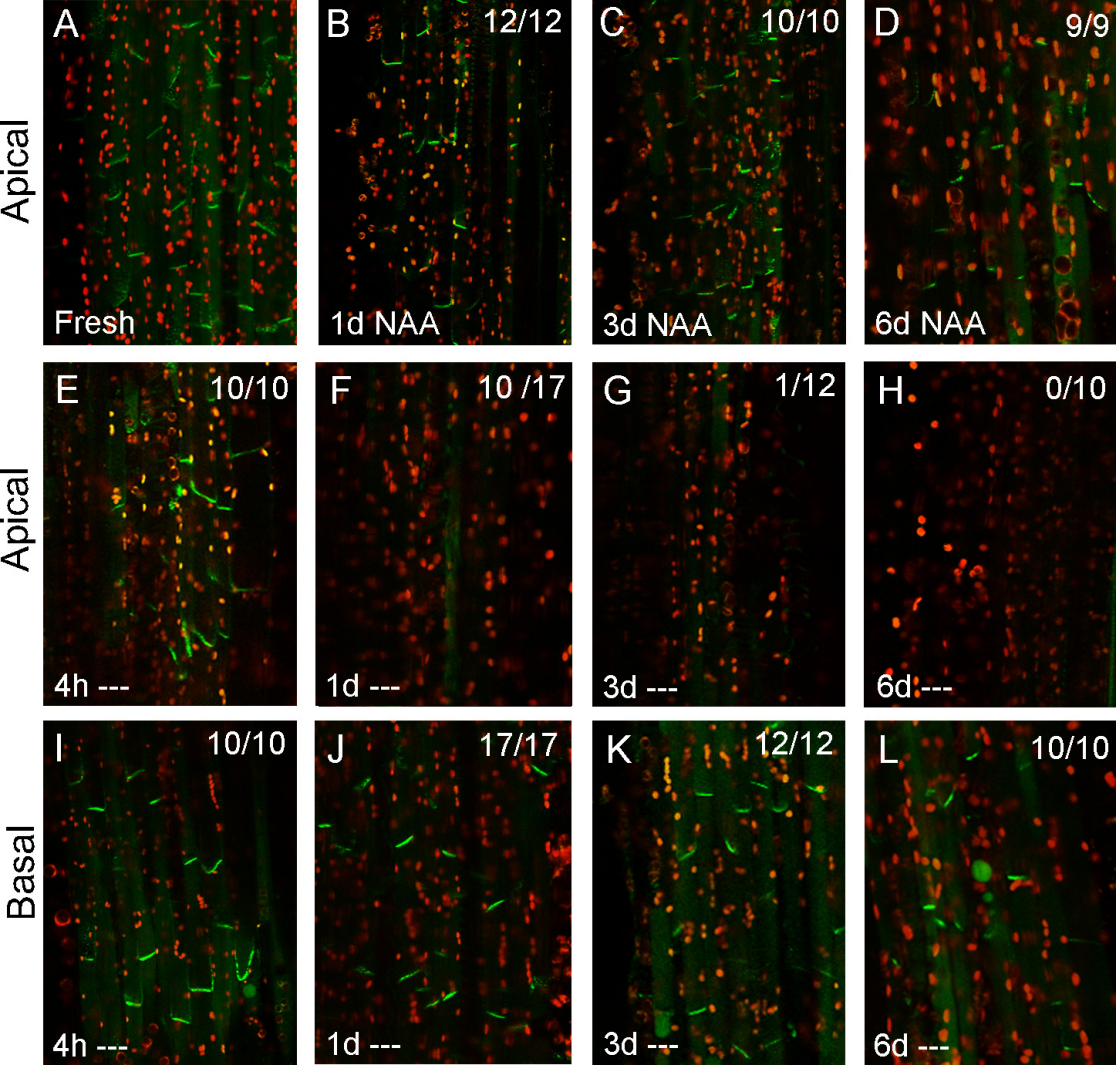
PIN1 dynamics in the stem are nonlinear. PIN1-GFP expression in xylem parenchyma cells (see Fig 1) in longitudinally hand sectioned ~2 cm basal inflorescence stem segments of 6-wk-old *PIN1pro*:*PIN1-GFP* plants. “Apical” and “basal” (at the left) refer to which end of the segment is being imaged. Numbers in the right hand corner indicate the number of segments/the number examined in which basal, polar PIN1 localization was seen in this treatment. Green signal indicates PIN1-GFP, red signal is chloroplast autofluorescence. **A)** Freshly harvested stem segments. **B,D)** Stem segments held vertically in Petri dishes between 2 agar blocks with 1 μM NAA supplied in the apical block for 1, 3, or 6 d, respectively. **E–L)** Stem segments held vertically in Petri dishes between 2 agar blocks with no hormone treatment (‘—‘) for 4 hours (E, I), 1 d (F, J), 3 d (G, K) or 6 d (H, L) at the apical end (E–H) or basal end (I–L) of the segment.

The 2 cm segments were held vertically between two sections of agar (after [40]) through which different treatments could be applied. Firstly, we assessed how PIN1-GFP responds to the absence of any such treatments (“untreated”), approximating the effect of decapitation of intact plants. We reasoned that, since the rate of auxin transport in stems is typically measured as ~6–10 mm/hour [41], any endogenous auxin present in these segments at the time of excision would be depleted within 4 hours. On the assumption that auxin flux maintains PIN1 polar localization, we therefore expected to observe rapid changes in PIN1 localization over this time-frame. However, we observed that PIN1 behavior is remarkably stable in this scenario. No obvious change in PIN1-GFP localization or expression occurred 4 hours after the segments were isolated (Fig 2A, 2E and 2I). These results suggest that either auxin depletion is not sufficient to trigger PIN1 endocytosis, or that auxin depletion has not occurred in these segments on the expected timescale.

After 1 d, PIN1-GFP on the basal plasma membrane was markedly reduced or absent at the apical end of many segments (7/17, Fig 2F). However, at the same time point, there was still strong basally localized PIN1-GFP in the medial (not shown) and basal parts of the stem segments (Fig 2J), and this signal persisted for up to 6 d after isolation, although gradually weakening (Fig 2K and 2L). In contrast, when we added 1μM naphthalene acetic acid (NAA), an auxin analog, to the apical block of agar (simulating an intact shoot apex), we observed that PIN1-GFP remained strongly expressed and polarized to the basal membrane throughout the stem segment for up to 6 d (Fig 2A–2D). Thus, auxin clearly promotes ongoing PIN1 localization at the basal plasma membrane of xylem parenchyma cells, consistent with the hypothesis that the presence of PIN1 at this location 4 hours after decapitation results from slower-than-expected auxin depletion. This hypothesis is further supported by the strong divergence in PIN1 behavior along the 2 cm segments, with an apical to basal progression in PIN1 depletion over 6 d, suggesting that rather than simple rapid basipetal auxin depletion, auxin transport dynamics in the stem are more complex.

### Stem Auxin Transport Kinetics Are Nonlinear

**Fig 3:**
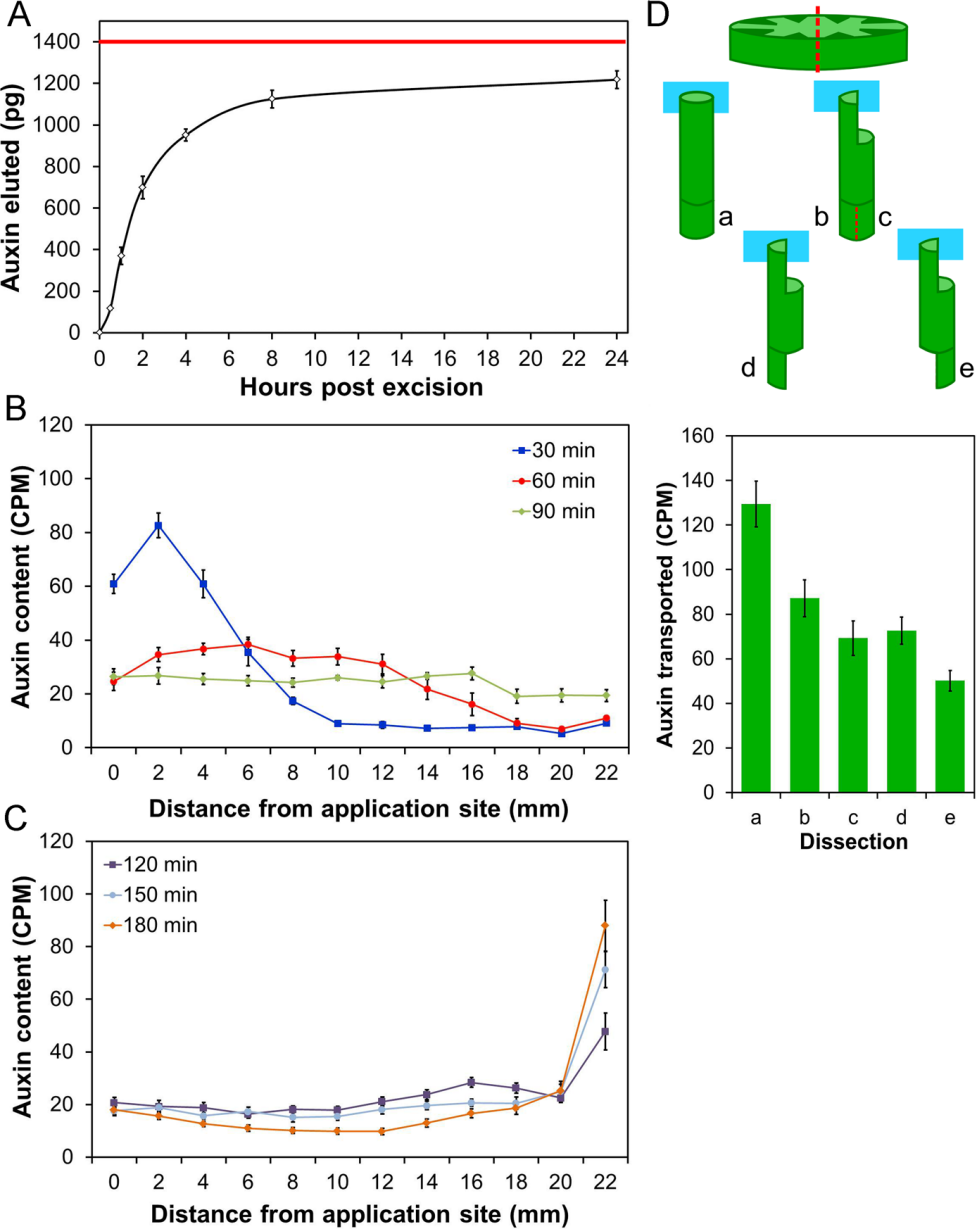
Auxin efflux dynamics from stem segments are nonlinear. **A)** Endogenous auxin eluted from 2 cm stem segments from the basal inflorescence internode of 6-wk-old plants was quantified by GC-MS at different points post excision. The cumulative auxin collected (pg) is shown relative to time. The red line indicates the approximate free auxin content of the stem segments at t = 0. **B)** Distribution of radio-labelled IAA (measured as CPM) in 2 mm intervals of 24 mm long stem segments after a 10 min pulse of 5 μM radio-labelled IAA was supplied to the apical end of the segment. Stems were dissected and analyzed by scintillation 30 min (blue line), 60 min (red line) or 90 min (green line) after the application of the pulse; *n* = 8 per time point, bars indicate standard error of the mean (s.e.m.). **C)** Distribution of radio-labelled IAA (measured as CPM) in 2 mm intervals of 24 mm long stem segments after a 10 min pulse of 5 μM radio-labelled IAA was supplied to the apical end of the segment. Stems were dissected and analyzed by scintillation 120 min (purple line), 150 min (light blue line) or 180 min (orange line) after the application of the pulse; *n* = 8 per time point, bars indicate s.e.m. **D)** Auxin transport assay to measure cross-stem auxin movement. Two schemes were trialed (b–c and d–e), in both of which the apical end of 18 mm stem segments were dissected so that radio-labelled auxin could be supplied to only half of the stem (see illustration). Longitudinal incisions were made across the diameter of the stem (top image, red dotted line), to a depth of 5 mm, followed by a second transverse incision to remove half the stem. Control segments (a) were left intact. In the d–e scheme, the basal end of the segment was similarly treated at the start of the experiment to leave either half the stem directly beneath the site of auxin application (d) or diametrically opposite the site of application (e), while in the b–c scheme the basal end was left intact during the assay. The apical end of the stem segments was then immersed in 2μM ^14C^IAA for 6 hours. At the end of the assay, the basal 5 mm of stem was dissected from the stems. In the b–c scheme, the basal 5 mm was longitudinally bisected, to separate the tissue directly under the site of auxin application (b) from the tissue diametrically opposite (c). The amount of radio-label transported into the basal 5 mm in each of a, b, c, d, and e was then measured by scintillation. The graph shows the auxin transported in each of these dissections (measured as CPM), *n* = 16, bars indicate s.e.m.

To test the idea that auxin levels in stem segments decline more slowly than we initially anticipated, we directly measured auxin levels in both the tissue and eluate of stem segments by gas chromatography-mass spectrometry (GC-MS). We found that freshly harvested basal stem segments contain on average 41 pg IAA per mg tissue (fresh weight) (standard deviation = 18, *n* = 4), equating to approximately 1,400 pg in a 20 mm segment (average mass ~35mg), providing a benchmark for total IAA at t = 0 (Fig 3A). We then assayed, also by GC-MS, the auxin eluted from the basal end of freshly harvested stem segments over a time-course, by serially transferring the segments to fresh collection buffer. In this way, we collected auxin in the time intervals 0–0.5 hours, 0.5–1 hours, 1–2 hours, 2–4 hours, 4–8 hours, and 8–24 hours. Almost half the auxin was collected in the first two hours, consistent with the rapid auxin depletion we expected from previously documented transport rates for auxin (Fig 3A). However, elution of auxin continued over the next 22 hours of the experiment, leading to an average cumulative eluate at 24 hours of 1218pg (standard deviation 42, *n* = 4); suggesting that by t = 24 hours approximately 10% of the original auxin content remained undrained (Fig 3A). However, when we analyzed tissue auxin content of stem segments that had been allowed to drain for 24 hours in the same manner, we found an average of 17.6 pg/mg IAA (standard deviation = 4, *n* = 4); equivalent to approximately 600 pg of IAA per segment (43% of the t = 0 content), consistent with net synthesis of ~400 pg IAA during the experiment. Taken together these data suggest that, in addition to a rapidly moving pool of auxin in the PATS, there may be a more slowly moving pool of auxin within stems and perhaps continued auxin synthesis. The combination of these factors appears to be sufficient to maintain PIN1 expression at the plasma membrane in the basal part of stem segments for many days following decapitation.

These data suggest that auxin transport in the stem may have complex kinetics. To test this hypothesis, we developed a pulse assay in which stem segments were inverted with their apical ends immersed in a solution of radio-labelled auxin for 10 min before being transferred to tubes containing media without auxin. We then tracked the distribution of radio-label within the segments over time, by cutting the segments into 2 mm sections and quantifying the radio-label present in each section. After 30 min, there was a relatively tight peak of radio-label towards the apical end of the segment, at a position consistent with most auxin molecules being transported at the commonly quoted rate of approximately 1 cm/h; however, a proportion of the auxin molecules in this assay moved substantially faster (Fig 3B). At subsequent time points (60 and 90 min), the auxin distribution became broader and shallower, and a distinct peak was difficult to discern (Fig 3B). Treatment with the auxin transport inhibitor 1-*N*-naphthylphthalamic acid (NPA) blocked the movement of auxin through these segments, showing that these profiles arose from active auxin transport (S1 Fig). From 120–180 min the radio-label gradually accumulated at the basal end of the stem, leaving only a very small residue across the rest of the stem (Fig 3C).

This rather diffuse pattern of auxin distribution is not consistent with auxin moving solely through the high conductance PATS. We hypothesized that this pattern arises because there is significant exchange between the PATS and the surrounding tissues [42]. The population of radio-labelled auxin molecules in this assay therefore does not move in a simple linear fashion though the transport stream, but is gradually spread along the stem and eventually collects at the base, moving on average more slowly than anticipated, but with a very large variance in the movement rate of the labeled auxin molecules.

### Auxin Moves across Stems

We have previously observed that two consecutive buds on an isolated stem segment can inhibit each other’s growth, despite connecting, and therefore exporting auxin, into different vascular bundles in the main stem [36]. In the context of slower and more complex than expected movement of auxin along the stem, we hypothesized that this inhibition might arise from auxin movement across the stem. To assess whether cross-stem auxin movement occurs we developed a cross-stem transport assay (Fig 3D), based on a modification of our basic bulk auxin transport assay in which stem segments are treated apically with radio-labelled auxin solution for 6 hours. For the cross-stem assay, radio-labelled auxin was supplied apically to only half the stem, and was then measured basally in either the same half, or the opposite half of the stem. If auxin moves strictly basipetally through the transport stream in each vascular bundle, there should be little radio-label collected in the opposite half, since within a single internode there is no vascular connection between the site of auxin application and collection [43]. However, in line with our hypothesis, a significant amount of auxin was detected on the opposite side of the stem to the site of auxin supply (Fig 3D). Taken together, these data suggest that, in addition to the classical PATS that is associated with vascular bundles, there is also appreciable auxin transport through a wide variety of tissues in the stem.

### *PIN1* Expression in the Stem Is Auxin Inducible but Highly Cell Type-Specific

**Fig 4:**
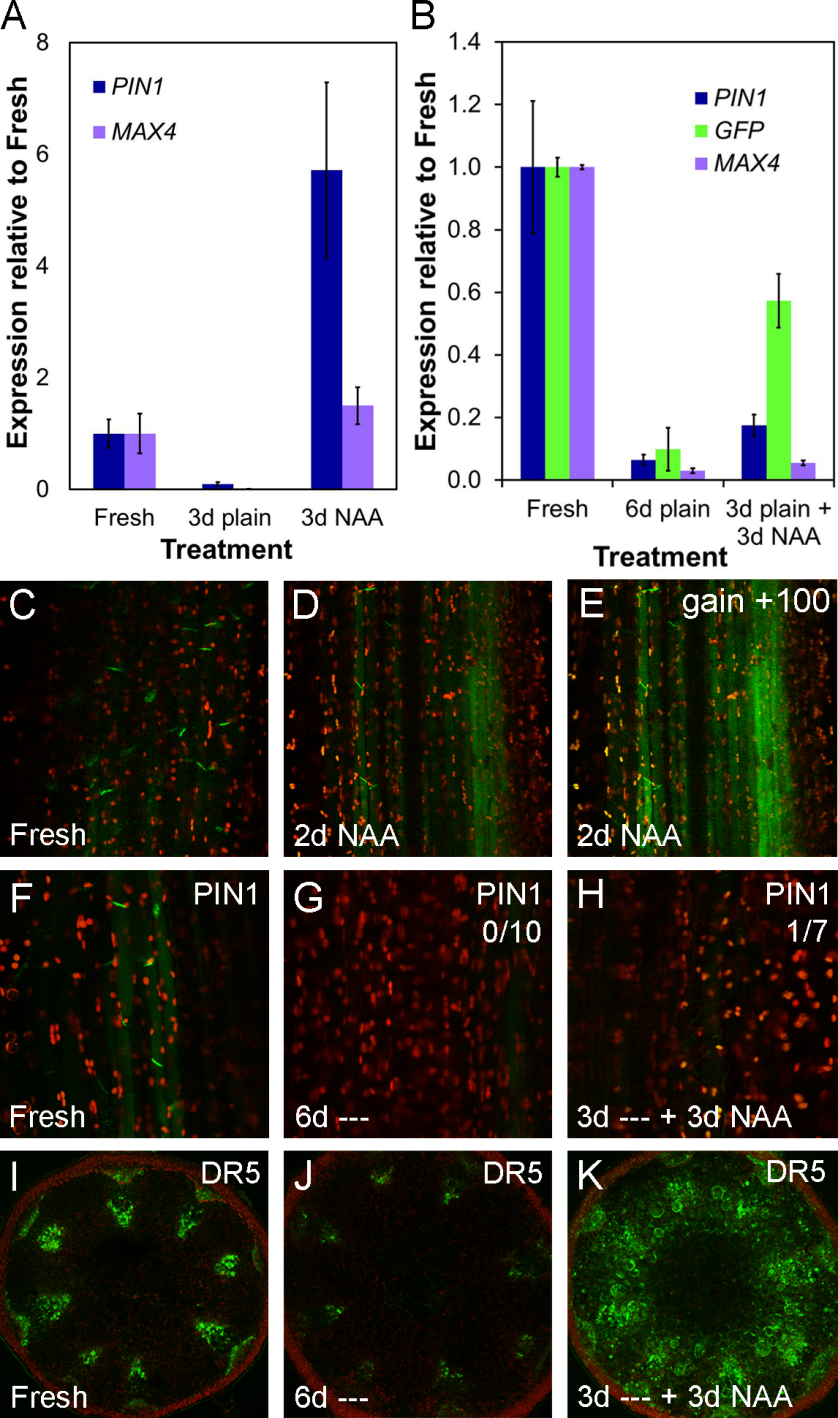
*PIN1* expression in the stem is auxin inducible but PIN1 accumulation is cell type-specific. **A)** *PIN1* and *MAX4* gene expression in ~2 cm stem segments from basal inflorescence internodes of 6-wk-old Col-0 plants freshly harvested, left untreated for 3 d or treated with 1 μM NAA for 3 d, as in Fig 2. Mean expression levels are shown relative to fresh segments and calculated from three biological replicates of 5–10 stems each using *UBC21* as a reference gene. Bars indicate s.e.m. **B)** *PIN1*, *MAX4* and *GFP* gene expression in ~2 cm stem segments from basal inflorescence internodes of 6-wk-old *DR5rev*:*GFP* plants freshly harvested, left untreated for 6 d or left untreated for 3 d and then treated for 3 d with 1 μM NAA as in Fig 2. Mean expression levels are shown relative to fresh segments, calculated from two biological replicates of 3–4 stems each using *UBC21* as a reference gene. Bars indicate s.e.m. **C–E)** PIN1-GFP expression in ~2cm stem segments from basal inflorescence internodes of 5-wk-old *PIN1pro*:*PIN1-GFP* plants, as in Fig 2, treated apically with 1 μM NAA for 2 d (D) relative to a freshly harvested stem (C). (E) shows the same image as D but with gain increased to show the maximum signal. Green signal indicates PIN1-GFP, red signal is chloroplast autofluorescence. **F–H)** PIN1-GFP expression in xylem parenchyma cells of ~2 cm stem segments from basal inflorescence internodes of 6-wk-old *PIN1pro*:*PIN1-GFP* plants; freshly harvested (F), left untreated for 6 d (‘—’) (G) and left untreated for 3 d and then treated with 1 μM NAA for 3 d (H). Numbers in the right hand corner indicate the number of segments/the number examined in which basal, polar PIN1 localization was seen in this treatment. Green signal indicates PIN1-GFP, red signal is chloroplast autofluorescence. **I–K)** GFP expression in ~2 cm stem segments from basal inflorescence internodes of 6-wk-old *DR5rev*:*GFP* plants; freshly harvested (I), left untreated for 6 d (‘—’) (J) and left untreated for 3 d, then treated with 1 μM NAA for 3 d (K).

The observed slow depletion of auxin suggests that the slow depletion of PIN1 (Fig 2) could reflect a canalization-like mechanism, with auxin flux being required to maintain PIN1 polarity. A further prediction of the canalization hypothesis is that auxin can induce expression of auxin transporters in naïve tissue, and indeed expression of the *PIN1* gene has previously been shown to be auxin-inducible in root and shoot apical meristems [44,45]. We therefore investigated whether the observed changes in *PIN1* expression in stem segments could be explained by changes in *PIN1* transcription. Using quantitative PCR, we analyzed transcription of *PIN1* in stem segments treated with apically applied 1μM NAA for 3 d, or left untreated for 3 d, compared to equivalent fresh stem segments. We found that *PIN1* transcription is induced approximately 6-fold by auxin treatment, and reduced approximately 10-fold in the absence of auxin (Fig 4A); expression of a control auxin-inducible gene, *MORE AXILLARY GROWTH4 (MAX4)*, behaved as expected in response to decapitation/auxin treatment [28]. However, when we analyzed GFP accumulation patterns in *PIN1pro*:*PIN1-GFP* plants in auxin treated segments, we did not observe any obvious change in the PIN1 expression pattern, even in very young stem segments taken immediately post-bolting. Cell files either expressed PIN1 or they did not, regardless of auxin treatment (Fig 4C–4E). This suggests that *PIN1* expression in the stem is auxin inducible but highly cell-type specific, and/or there is cell-type specific post-transcriptional regulation.

To examine further the auxin inducibility of *PIN1* expression in the stem, we left *PIN1pro*:*PIN1-GFP* stem segments untreated for 3 d so that PIN1 was depleted from the basal plasma membranes of xylem parenchyma cells at the apical end of the stem segment. We then treated them with apically applied 1μM NAA for 3 d. We expected to see recovery of *PIN1* expression, but these stem segments had very low expression levels of PIN1-GFP after the treatment, comparable to stem segments left untreated for 6 d, and unlike stems treated for 6 d with NAA (Fig 4F–4H, Fig 2D). In contrast, expression of the well-established auxin-inducible *DR5rev*:*GFP* transgene [46] was strongly induced in the majority of cell types in stem segments subjected to the same treatment (Fig 4I–4K). We performed quantitative PCR on RNA extracted from *DR5rev-GFP* stem segments used in this experiment, and observed changes in *GFP* transcript abundance in line with our visualization of GFP expression (Fig 4B). However, *PIN1* transcription in these stems responded only very weakly to auxin treatment after 3 d left untreated (Fig 4B), suggesting that the auxin-inducibility of *PIN1* is conditional, and that in auxin-depleted stems, *PIN1* expression is transcriptionally locked out, an effect also seen with *MAX4* in this experiment (Fig 4B). Taken together, these data suggest that PIN1 accumulation in the stem is cell-type specific, and that PIN1 protein levels in the basal plasma membrane of xylem parenchyma cells do not correspond reliably to *PIN1* transcript abundance.

### Auxin Concentration Maintains PIN1 Localization at the Membrane

The behavior of PIN1 in untreated stem segments described above is consistent with auxin acting to keep PIN1 localized to the basal plasma membrane of xylem parenchyma cells. In roots, auxin has been shown to prevent removal of PIN1 protein from the membrane in a transcription-independent fashion [47]. Feedback-regulation of PIN1 protein by auxin is a well-established concept in plant development (reviewed in [48]), and allocation of PIN1 in proportion to the flux across a given membrane (“with the flux”) is integral to most canalization models [48]. We therefore tested whether auxin flux itself is an important component of PIN1 regulation in stems. To test this, we used NPA, which strongly inhibits auxin transport in stem segments (e.g., [10], S1 Fig), and causes apical accumulation of applied auxin (S1 Fig).

**Fig 5:**
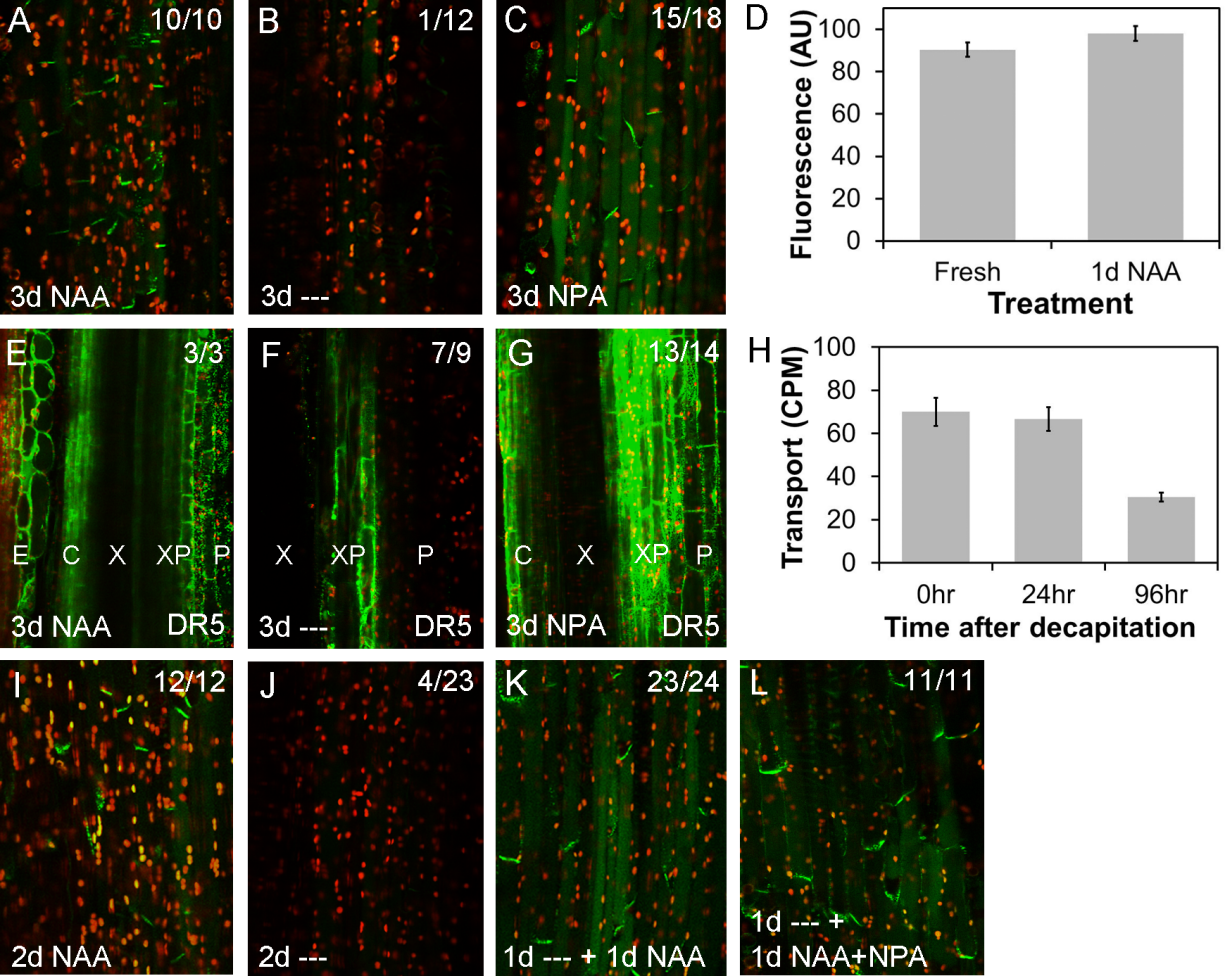
Auxin promotes PIN1 localization at the plasma membrane. For confocal images (A–C; E–G, I–L), green signal indicates GFP, red signal is chloroplast autofluorescence. Images are from hand-cut sections of ~2 cm basal inflorescence internode segments from of 6-wk-old plants, held vertically between agar blocks for the times indicated and imaged at the apical end of the segment. **A–C)** PIN1-GFP expression in xylem parenchyma cells of *PIN1pro*:*PIN1-GFP* segments treated apically with 1 μM NAA for 3 d (A), no treatment (‘—’) for 3 d, (B) or 1 μM NPA for 3 d (C). Numbers in the right hand corner indicate the number of segments/the number examined in which basal, polar PIN1 localization was seen in this treatment. **D)** Quantification of PIN1-GFP levels (in arbitrary units, AU) at the basal plasma membrane in freshly harvested *PIN1pro*:*PIN1-GFP* stem segments versus segments treated apically for 1 d with 1 μM NAA. Forty-five membranes were analyzed per treatment (five each of nine individual stem segments); bars represent s.e.m. **E–G)** GFP expression in *DR5rev-GFP* stem segments treated apically with 1 μM NAA for 3 d (E), no treatment (‘—’) for 3 d, (F) or 1 μM NPA for 3 d (G). Numbers in the right-hand corner indicate the number of segments/the number examined in which the pattern shown was observed. **H)** The ability of basal internode segments 0, 24, and 96 hours after excision from the plant to transport radio-labelled IAA to their basal 5 mm (measured as counts per minute, CPM) during a 6 hour incubation with their apical ends immersed in radio-labelled auxin. The segments were held vertically between agar blocks for 0, 24, and 96 hours, as in A–G, before transfer to Eppendorf tubes for the auxin transport assay. *n* = 14–16; bars indicate s.e.m. **I–L)** PIN1-GFP expression in xylem parenchyma cells of *PIN1pro*:*PIN1-GFP* segments treated apically with 1 μM NAA for 2 d (I), no treatment (‘—’‘) for 2 d, (J) or no treatment for 1 d followed by 1 μM NAA for 1 d (K), or 1 μM NAA and 1 μM NPA for 1 d (L). Numbers in the right hand corner indicate the number of segments/the number examined in which basal, polar PIN1 localization was seen in this treatment.

Cells at the apical end of stems treated apically with 1 μM NPA for 3 d maintain PIN1 localization in their basal plasma membranes (Fig 5A–5C) and have higher *DR5-GFP* expression than untreated stem segments (Fig 5E–5G). This suggests that the accumulation of auxin caused by the inhibition of auxin transport is sufficient to maintain the polar localization of PIN1 in the apical end of stem segments. Conversely, since auxin flux is inhibited in these segments, a direct flux-based mechanism cannot account for the maintenance of PIN1 localization. Our data suggest that maintenance of PIN1 localization in stem xylem parenchyma cells, at least over the 3 d timeframe of this experiment, is driven by a concentration-dependent and flux-independent mechanism.

Another central aspect of the canalization hypothesis is that PIN1 accumulation at the membrane should increase and polarize in response to auxin. To test this idea, we assessed whether treatment with 1 μM NAA for 1 d could increase basal plasma membrane PIN1 accumulation, but we found no significant change relative to freshly harvested segments (*t* test, *n* = 9, *p* = 0.167) (Fig 5D). These data suggest that either membrane levels of PIN1 in the stem are normally saturated, or that PIN1 levels do not respond to auxin. To bring PIN1 levels below a hypothetical saturation point, we left stem segments untreated for 1 d, before applying 1 μM NAA apically for 1 d. We observed strong PIN1 localization on basal plasma membranes (Fig 5K) compared to control segments left untreated for 2 d, in which PIN1 was undetectable (Fig 5I–5K). Again, we tested whether auxin flux itself was important in this process, by applying 1 μM NPA together with 1 μM NAA for 1 d to stems previously untreated for 1 d. NPA had no effect on the re-accumulation of PIN1 at the basal plasma membrane (Fig 5L). Taken together, these data suggest that auxin can indeed promote (re-)allocation of PIN1 to the membrane, but this does not depend directly on auxin flux.

Recent results demonstrate that PIN1 phosphorylation can modulate its activity, providing a possible mechanism for auxin to regulate its own transport, independent of both transcription and plasma membrane levels of PIN1 protein [49]. We have previously observed a close correlation between PIN1 levels in the basal plasma membrane of xylem parenchyma cells in stems and bulk polar auxin transport through stem segments [12,34]. To assess this correlation in the context of auxin depletion following decapitation, we compared auxin transport in freshly harvested stem segments to those 1 d and 4 d following their excision. We observed a strong correlation between auxin transport and PIN1 levels, with transport reducing over time in concert with levels of PIN1 at the basal plasma membrane (Fig 5H).

### Modelling of Auxin Transport Supports a Multi-modal System

Our data show that auxin transport dynamics in mature stem segments are more complex than we anticipated, and furthermore PIN1 expression in these segments appears to be tissue specific and does not behave in a classically canalization-like manner. This contrasts with PIN1 behavior in organ primordia and developing leaves, which appears more consistent with the canalization hypothesis [50,51].

**Fig 6:**
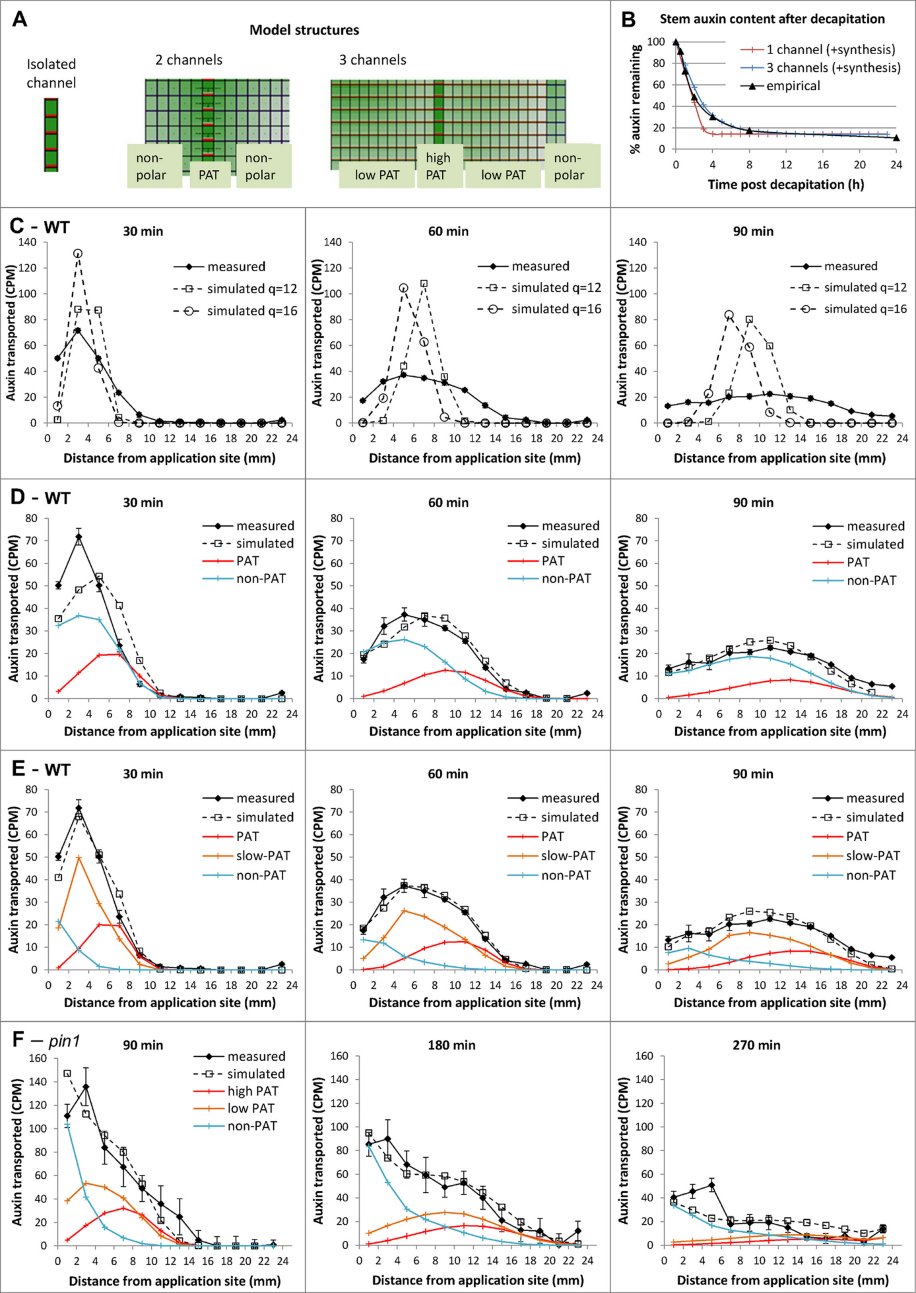
Multi-channel models of auxin transport capture observed stem dynamics. **A)** The three types of grid structure used to represent tissue organization in the single and multi-channel models. In all three models, cells have dimensions 0.1 mm x 0.01 mm; auxin diffusion rate: D = 16e-3 mm^2^/min. **B)** Computer simulations compared to empirical data of the drainage of auxin in an excised stem segment; parameter values are as in C and E, with additional synthesis rates: 3×10^−5^ auxin units /min^-1^ (single channel model), 3.8×10^−6^ auxin units/min^-1^ (three-channel model). The empirical data are as in Fig 3A. **C–E)** Computer simulations compared to empirical data of the auxin pulse assay in WT using the single channel model (C), the two-channel model (D), and the three-channel model (E). For the measured data, experiments were as described in Fig 3B and 3C. Error bars show the s.e.m., *n* = 8 per time point. **F)** Same simulation as in E, but for the *pin1* mutant and later time points. For the measured data, experiments were as described in Fig 3B and 3C, but using *pin1-613*. Error bars show the s.e.m., *n* = 8 per time point. Parameter values (mm/min): **C.** *p* = 16, q as shown on graph; **D.** high conductance polar channel: p_1_ = 6, q_1_ = 0.1; q_12_ = 0.; non-polar channel: q_3_ = 0.01, q_32_ = 0.01; **E.** high conductance polar channel: p_1_ = 2, q_1_ = 0.2; q_12_ = 9 10^−4^; low conductance polar channel: p_2_ = 0.3, q_2_ = 0.7, q_23_ = q_22_ = q_21_ = 0.01; non-polar channel: q_3_ = 0.3, q_32_ = 2.5 10^−4^; **F.** high conductance polar channel: p_1_ = 0.5, q_1_ = 0.2; q_12_ = 9 10^−4^; low conductance polar: p_2_ = 0.075, q_2_ = 0.7, q_23_ = q_22_ = q_21_ = 0.01; non-polar channel: q_3_ = 0.3, q_32_ = 2.5 10^−4^.

To deepen our understanding of these processes, we developed a computational framework to assess the requirements for generating the auxin transport dynamics we observed (described in S2 Fig). First, we represented auxin transporting tissues in the stem by an isolated file of rectangular cells, as shown in Fig 6A. The model allows auxin to diffuse along the length of the cell, and to cross into a neighboring cell with a rate dependent on membrane permeability (transporter activity). The apoplast (cell wall space) is not explicitly represented. We call the two opposite ends of the cell “apical” and “basal.” To match plant stems, we orientated auxin transport basipetally, and gave the basal membrane a polar permeability component *p*, in addition to the non-polar component *q*, which is common to both apical and basal membranes. Auxin thus exits a cell with rate *p+q* at the basal end and with rate *q* at the apical end, as in [42] and [52]. We then simulated the pulse assay described above (Fig 3C and 3D) and found that for the range of values of the non-polar component *q* that would allow the pulse to travel at the observed velocity, the pulse profile maintained a narrow peak over time, which progressed all the way down the stem section. This poorly matches the broadening, flattening profiles obtained experimentally in Arabidopsis stems (Fig 6C).

We then modelled an alternative scenario, in which the PATS is not considered in isolation but rather as part of a more complex transport system. We used a grid of similarly shaped cells to represent a longitudinal section through the stem, with tissues—or channels—with specific auxin transport properties. In its simplest form, the grid was split into two channels: polar and non-polar (Fig 6A). As in our first model, the polar channel cells have basal permeability *p*_*1*_ *+q*_*1*_ and apical permeability *q*_*1*_; they now also have lateral permeability *q*_*12*_. In the non-polar channel, cells have identical basal and apical permeabilities *q*_*2*_ and lateral permeability *q*_*21*_. We simulated the pulse assay again, with model parameters fitted manually to the data, and found that this two-channel structure was sufficient to reproduce the flattening over time characteristic of experimental profiles (Fig 6D), with the polar stream effectively dragging a mass of auxin spread out in non-polar tissue. The narrow peak shown on the 30 min pulse, however, was still imperfectly captured by the simulation. We observed the same results if we used parameters obtained by automated parameter search, using the Nelder-Mead simplex algorithm (GNU Scientific Library). This automated fitting was performed by initializing to random parameter values, with certain constraints on the initial parameter range required to ensure lateral permeabilities were at least an order of magnitude smaller than vertical ones (fully described in S1 Text).

Another grid structure was then implemented along the same principles, but with three channels: high-conductance polar (equivalent to the PATS), low-conductance polar and non-polar. In this case, the low-conductance channel is positioned between the high-conductance polar channel and the non-polar channel (Fig 6A). Simulations showed that this three-channel structure was able to reproduce closely the shape of the average empirical profiles (Fig 6E). A multi-channel structure (either two- or three-channel) implementing mobility of auxin both within and between the different channels therefore seemed key to capturing the essential aspects of observed auxin pulse progression in Arabidopsis stems. Recent work, both computational and analytical, investigates possible causes for pulse profile flattening in other plant species and draws a similar conclusion [42]. Furthermore, having manually fitted the three-channel auxin transport model to Arabidopsis pulse data, with the same parameters we were able to reproduce the slower than expected auxin drain profile in excised stems, with the inclusion of a tissue-wide basal auxin synthesis term that was absent in the pulse simulations (Fig 6B). This model is therefore robust to scenarios in which stems are fully loaded with auxin.

We explored the three-channel model further, using it to investigate the effect of *pin1* mutations on pulse shape. The results of these simulations compared to the measured transport profiles in *pin1* mutants are presented in Fig 6F and S3 Fig. We found that simulating reduction of polar transport by altering the polar component of membrane permeability in the high-conductance channel alone (*p1*) resulted in reduced movement of the auxin front, but did not dramatically slow movement of the body of the pulse (S3 Fig). If we reduced the polar component of membrane permeability in the two polar channels of the stem (*p*_*1*_ and *p*_*2*_), we observed the same slower moving front of auxin, together with retention of the main body of auxin at the apical end of the stem (S3 Fig). When we measured auxin pulses in the *pin1-613* mutant over equivalent time-frames, the pulse shapes matched those predicted by altering both *p1* and *p2* (Fig 6E and 6F). In particular, we found that pulses across multiple time points in *pin1-613* closely match simulations in which *p*_*1*_ and *p*_*2*_ are reduced by 4-fold, (Fig 6F). We also simulated a bulk auxin transport assay using the same parameters and found that *pin1* was predicted to have 27% of the auxin transport in wild-type, which is highly consistent with previous measurements of auxin transport in *pin1* [12] (S3 Fig). The three-channel model is thus able to incorporate perturbations to the system and, in this specific example, predicts that PIN1 is important in both high- and low-conductance polar auxin transport.

### Auxin Transport Proteins Are Expressed in Diverse Stem Tissues

**Fig 7:**
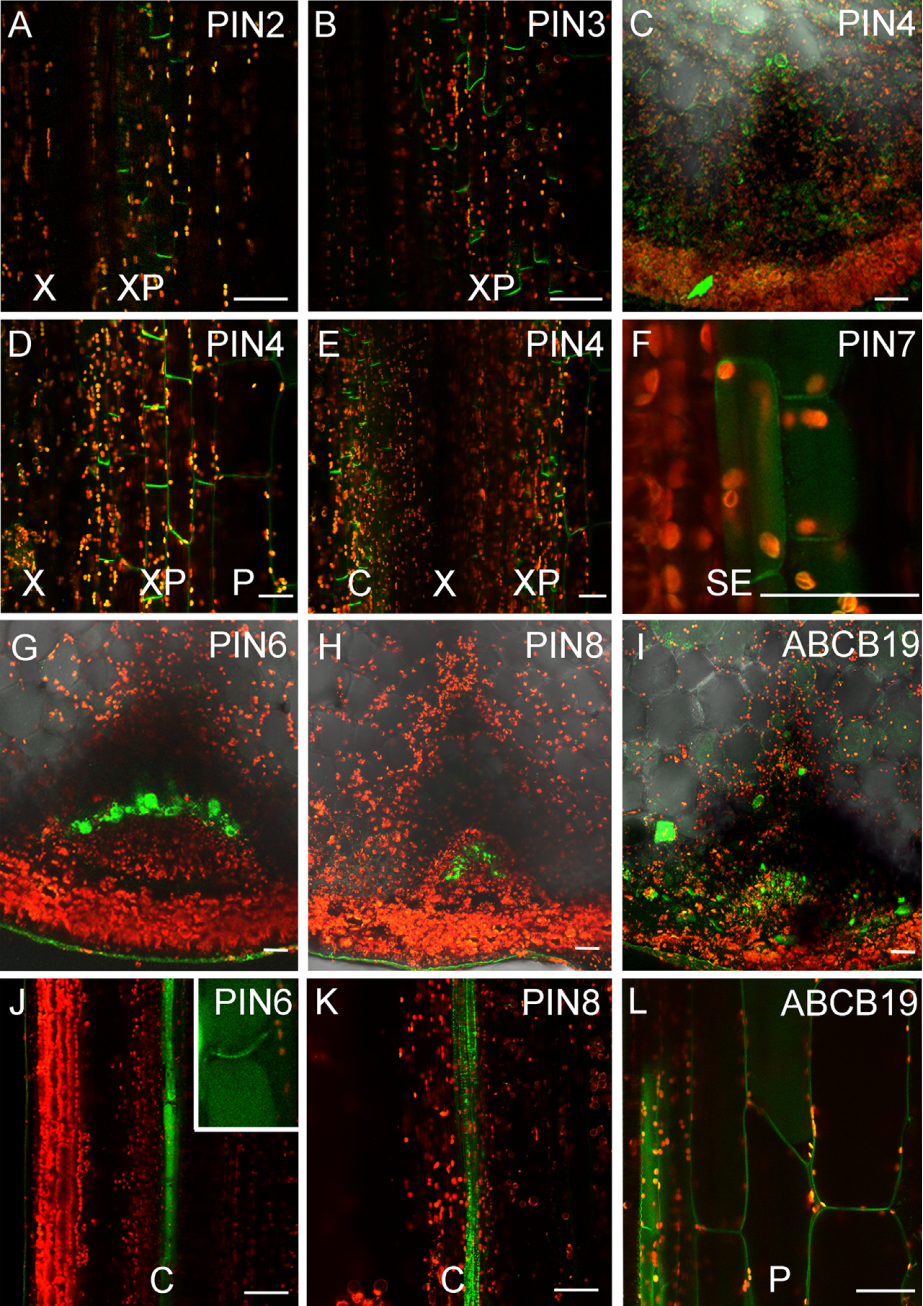
Broad expression of auxin transporters in stem tissues. Expression of fluorescent protein fusions of auxin transporters in transverse or longitudinal hand sections of basal inflorescence stem internodes from 5- or 6-wk-old plants. Patterns were consistent amongst all individuals examined, *n* > 10 for each line. Scale bars indicate ~30 μm. **A)** *PIN2*:*PIN2-GFP* expression in the xylem parenchyma, longitudinal section. **B)** *PIN3*:*PIN3-GFP* expression in the xylem parenchyma, longitudinal section. **C–E)** *PIN4*:*PIN4-GFP* expression in pith, xylem parenchyma and cambium cells, transverse (C) and longitudinal sections (D,E) of 5-wk-old stems. **F)** *PIN7*:*PIN7-GFP* expression in sup-epidermal cells, longitudinal section of 5-wk-old stem. **G,J)** Expression of *PIN6*:*PIN6-GFP* in basal internodes in transverse (G) and longitudinal (J) cross sections. Inset shows plasma membrane-localized PIN6-GFP in addition to probable ER-localized signal. **H,K)** Expression of *PIN8*:*PIN8-GFP* in basal internodes in transverse (H) and longitudinal (K) cross sections. **I,L)** Expression of *ABCB19*:*GFP-ABCB19* in basal internodes in transverse (I) and longitudinal (L) cross sections. C = Cambium, P = Pith, SE = Sub-epidermis, X = Xylem, XP = Xylem parenchyma.

**Fig 8:**
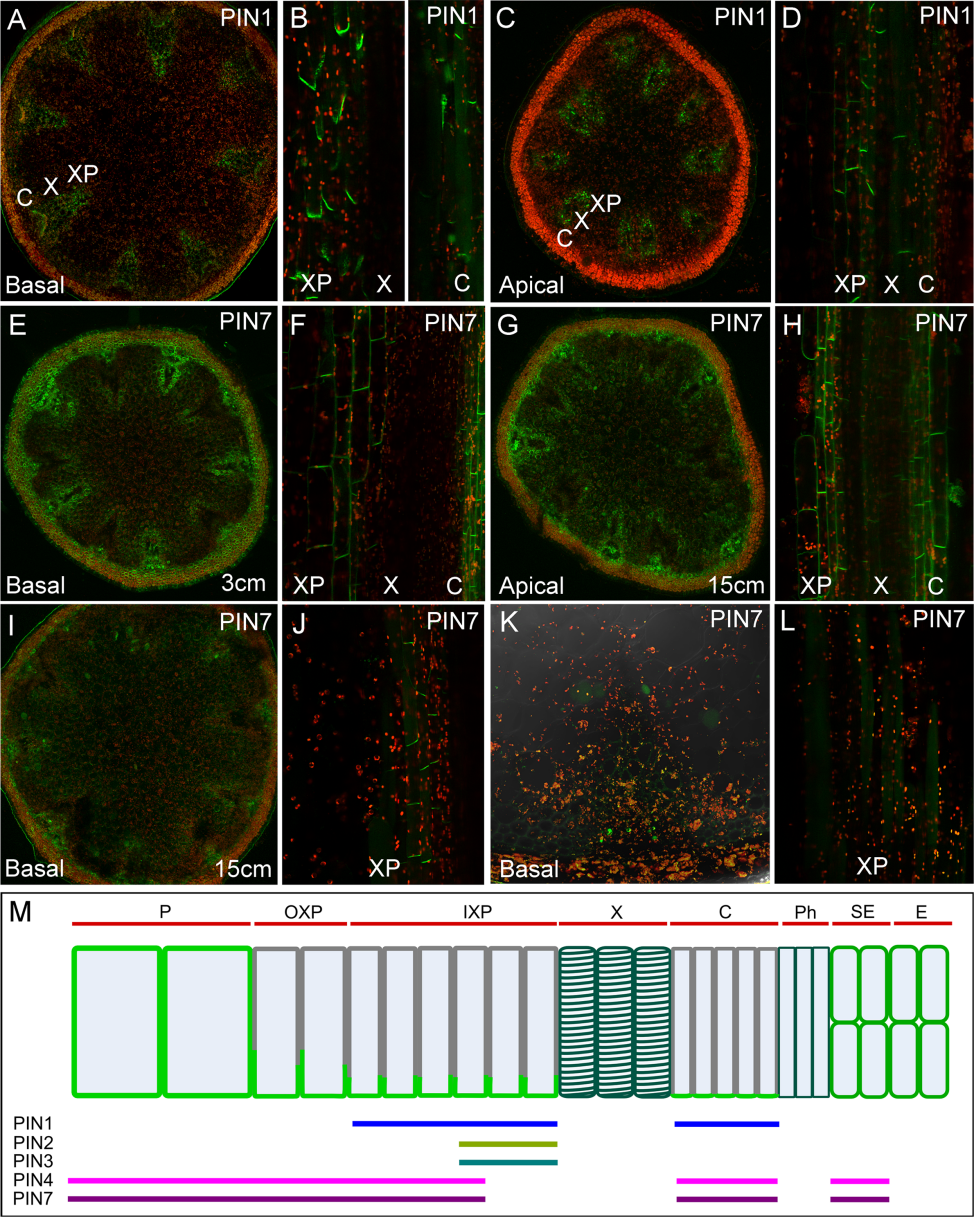
*PIN7* expression is dynamic during stem development. **A–D)** *PIN1*:*PIN1-GFP* expression in the cambium and xylem parenchyma, transverse (A,C) and longitudinal sections (B,D) of basal (A,B) and apical (C,D) internodes of 30cm tall (= 6/7 wk old) inflorescence stems. **E–F)** *PIN7*:*PIN7-GFP* expression in transverse (E) and longitudinal sections (F) of basal internodes of 3 cm tall (= 5 wk old) inflorescence stems. **G–J)** *PIN7*:*PIN7-GFP* expression in transverse (G,I) and longitudinal sections (H,J) of basal (G,H) and apical (I,J) internodes of 15 cm tall (= 5/6 wk old) inflorescence stems. **K–L)** *PIN7*:*PIN7-GFP* expression in transverse (K) and longitudinal sections (L) of basal internodes of 30 cm tall (= 6/7 wk old) inflorescence stems. **M)** Summary of efflux carrier expression in Arabidopsis stems, showing PIN expression domains and localization patterns in a simplified longitudinal transect of cells in a vascular bundle and surrounding tissue. Tissue domains are indicated by red bars at the top. Expression domains of PIN1, PIN2, PIN3, PIN4, and PIN7 are indicated by colored bars at the bottom. ABCB19 is expressed in all tissues except the xylem and phloem (not shown). PIN proteins are not expressed (at least at detectable levels) in xylem or phloem vessels. PIN localization in cells is indicated by light green. PIN localization is apolar in pith and sub-epidermal cells and polar in other cell types, irrespective of the PIN protein type.

The results from our models suggest the existence of multiple, spatially distinct transport regimes in the stem. In order to examine this idea further, we analyzed expression and localization patterns of auxin efflux carriers in the stem, since efficient intercellular movement of auxin has generally been considered to be dependent on their activity. As discussed above, *PIN1*:*PIN1-GFP* expression is largely restricted to the xylem parenchyma and cambial cells, tissues classically associated with the PATS (Fig 3J) [10,20]. We observed very weak expression of *PIN2*:*PIN2-GFP* in the xylem parenchyma, while *PIN3*:*PIN3-GFP* is also expressed primarily in the xylem parenchyma cells (Fig 7A and 7B). In contrast, *PIN4*:*PIN4-GFP* and *PIN7*:*PIN7-GFP* have much broader expression patterns in young bolting stems (0–15 cm tall), but expression gradually declines as the tissue matures (Fig 7C–7F; Fig 8E–8L; S4 Fig). PIN4 and PIN7 are expressed in cambium, and in a broader set of xylem parenchyma cells than PIN1, as well as pith and epidermal cells (Fig 7D–7F; Fig 8E–8L, S4 Fig). *PIN6*:*PIN6-GFP* is expressed in a single stripe of cambium cells directly adjacent to the xylem (Fig 7G and 7J). As previously reported, a lot of PIN6-GFP signal is located in internal cell compartments [53], but—consistent with its semi-canonical structure—we also observed PIN6 localized at the plasma membrane in a bipolar (or possibly apolar) manner (Fig 7J). *PIN8*:*PIN8-GFP* is expressed in a narrow band of cambial cells located outside the band of cells expressing PIN6 (Fig 7H and 7K). Consistent with previous reports, PIN8 seems to be localized to intracellular compartments [53]. Members of the ATP-BINDING CASETTE (ABC) B family of transporters also function in auxin efflux in Arabidopsis [53,54], and we thus examined expression of an *ABCB19*:*GFP-ABCB19* fusion protein [56]. ABCB19 has the broadest expression pattern of the proteins genes we examined, and can be observed with an apolar localization in the majority of cells in the stem with the exception of xylem and phloem (Fig 7I and 7L). This is consistent with both the inferred function of ABCB19 in generalized auxin efflux [57], and with our data showing that almost all apically applied auxin eventually emerges from stem segments at the basal end (Fig 4B and 4C). We thus conclude that, although expression of transporters is unsurprisingly highest in the well-established PATS tissues, almost all cells in the stem have the ability to mobilize auxin.

### PIN7 Expression Is Dynamic during Stem Development

We were particularly intrigued by the expression of PIN4 and PIN7 (Fig 8) surrounding the classic PATS tissues, which might support the idea of a third transport regime in the stem. *PIN7*:*PIN7-GFP* expression is consistently stronger than *PIN4*:*PIN4-GFP*, so we decided to focus on analysis of *PIN7* expression. In the basal internodes of very young, 3 cm tall inflorescences, *PIN7* is expressed in most cells in the stem (Fig 8E). In the xylem parenchyma and cambium, PIN7-GFP is localized to all cell faces, though it appears to be strongest at the basal membrane (Fig 8F). However, in the basal internodes of older, 15 cm tall inflorescences, *PIN7* expression is more restricted. It cannot be detected in xylem parenchyma cells directly adjacent to the xylem, which express *PIN1* (compare Fig 8A and 8I), but is expressed at relatively high levels in xylem parenchyma cells nearer the pith (the “outer” xylem parenchyma) (Fig 8I). Its localization in these cells appears fully polarized (Fig 8J). *PIN7* is still expressed in the pith and sub-epidermal cells at this stage, in which it remains apolar (Fig 8H). In the basal internodes of 30 cm tall inflorescences, PIN7-GFP cannot be detected at the plasma membrane in any cell, though expression in an intracellular compartment is sometimes seen (Fig 8K and 8L). However, in the apical internodes of older inflorescences, *PIN7* expression is still strong, and its expression and localization resembles that seen in very young basal internodes (Fig 8G and 8H). *PIN4* expression is very similar to *PIN7*, and it shows the same general pattern of changes in expression as the stem ages (S4 Fig). *PIN4* and *PIN7* thus display dynamic age/stage-specific behavior during stem development. In young internodes they are expressed broadly and less polarly, and gradually become more restricted and more polar as those internodes mature.

### ABCB1 and ABCB19 Contribute to Stem Auxin Transport

Our results show that different tissues in the stem accumulate different complements of auxin efflux carriers with different localization patterns (Fig 8M). This is consistent with the predictions of our models, and suggests that multiple, spatially-distinct auxin transport regimes exist within the stem. Cells in the cambium and xylem parenchyma, which are known to perform high-conductance polar transport (i.e., the PATS) have highly polarized expression of PIN1, along with PIN2 and PIN3. Cells in the pith and epidermis have only apolarly-localized efflux carriers, and thus are likely to correspond broadly to the non-polar channel in our model. Finally, cells located between the pith and xylem parenchyma have less-polarly localized expression of PIN4 and PIN7, at least in young stems, and may therefore broadly correspond to the low-conductance polar channel in our model. Their overlapping expression patterns suggest that each efflux carrier can contribute to multiple regimes; the regimes are thus tissue-specific, not transporter-specific.

**Fig 9:**
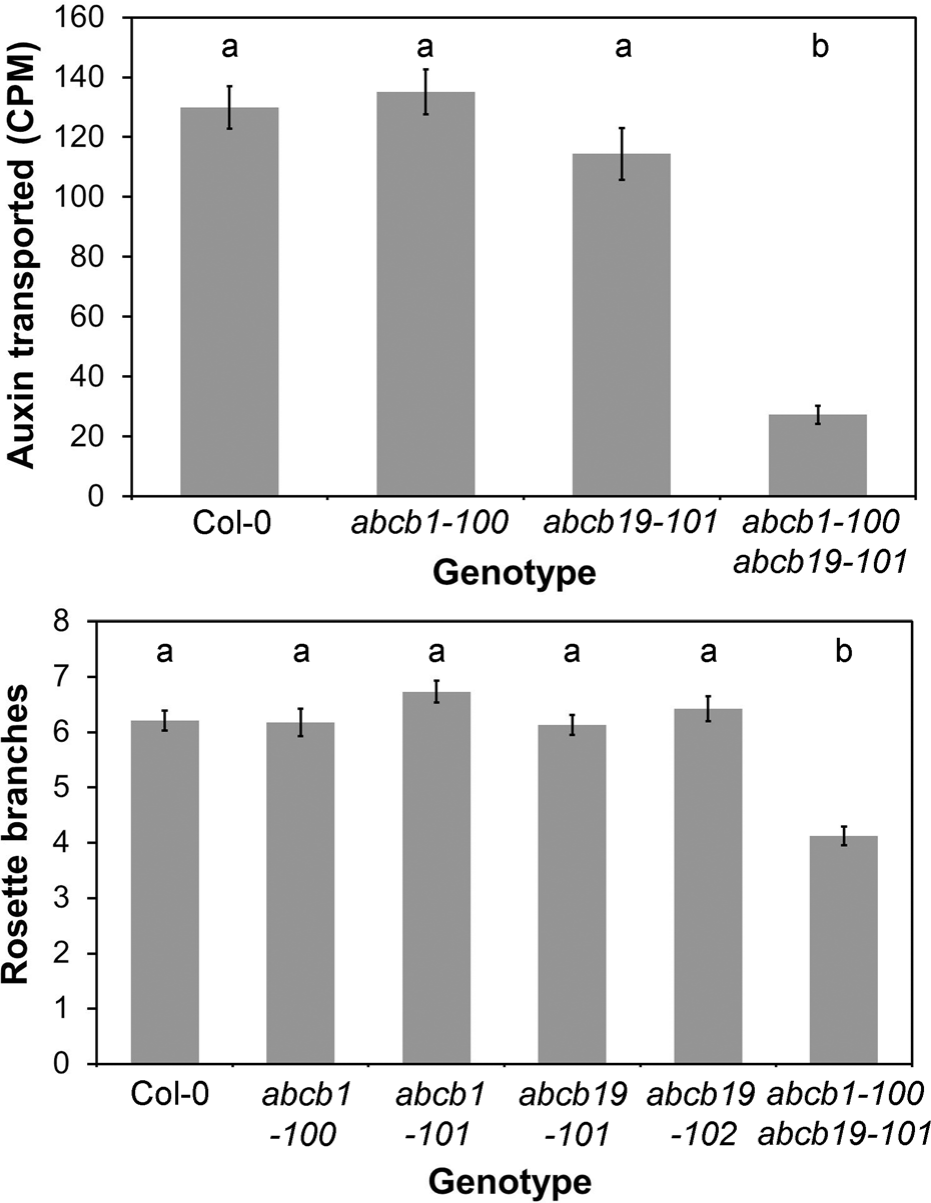
ABCB1 and ABCB19 contribute to stem auxin transport **A)** Bulk basipetal auxin transport (measured in CPM) in the basal internodes of 6-wk-old Col-0, *abcb1-100*, *abcb19-101* and *abcb1-100 abcb19-101* plants. *n* = 24; bars indicate s.e.m. Bars with different letters are significantly different from each other (ANOVA, Tukey HSD, *p* < 0.05). **B)** Rosette branching in Col-0, *abcb1-100*, *abcb1-101*, *abcb19-10*,*1 abcb1-102*, and *abcb1-100 abcb19-101* 10 d after decapitation. *n* = 15–24; bars indicate s.e.m. Bars with different letters are significantly different from each other (ANOVA, Tukey HSD, *p* < 0.05).

We next aimed to test whether the contributions to auxin movement from these apparent transport regimes is consistent with the predictions of our model. We have already shown that severely reducing high-conductance transport through loss of the PIN1 protein greatly retards the movement of auxin in the stem (S3 Fig). To test the possible role of the non-polar regime, we examined mutants in *ABCB19*, which is the most highly expressed carrier in pith cells, and its redundantly acting homologue *ABCB1*. We reasoned that although ABCB19 is expressed broadly in the stem, its loss might particularly affect the non-polar regime. We examined several previously described *abcb1* and *abcb19* single mutants, but did not observe any reproducible effect on auxin transport in these lines (Fig 9A). Consistent with previous reports, we found that the *abcb1-100 abcb19-101* double mutant has a very severe developmental phenotype with extreme dwarfing, and has dramatically reduced auxin transport (Fig 9A) [54]. The phenotype of *abcb1 abcb19* makes it very difficult to interpret the auxin transport data meaningfully. The reduced transport could arise directly from loss of transport proteins in the stem, or altered stem anatomy, or both. More generally, the comparability of the values obtained for the double mutant vs other genotypes is compromised by the fact that the internodes of the double mutant are so short that it is only possible to assess transport across multiple nodes, in contrast to the intermodal segments used for other genotypes.

With respect to shoot branching, the *abcb1 abcb19* double mutant has previously been characterized as having a bushy phenotype [54]. However, we did not observe any increase in shoot branching in the *abcb1-100 abcb19-101* double mutant or in any of the *abcb1* or *abcb19* single mutants we examined (Fig 9B). Indeed, branching in *abcb1-100 abcb19-101* is actually reduced relative to wild type, though given the severe developmental phenotype, caution is also required in interpreting these data. The previously reported bushiness of *abcb1 abcb19* is a very late developmental effect (see details in [54]), probably caused by the infertility of the double mutant. Overall, we conclude that phenotypes of the double mutant are too pleiotropic to allow detailed assessment of the role of the non-polar auxin transport regime in auxin movement or shoot branching.

### PIN3, PIN4, and PIN7 Mediate Auxin Transport Connectivity in Stems

**Fig 10:**
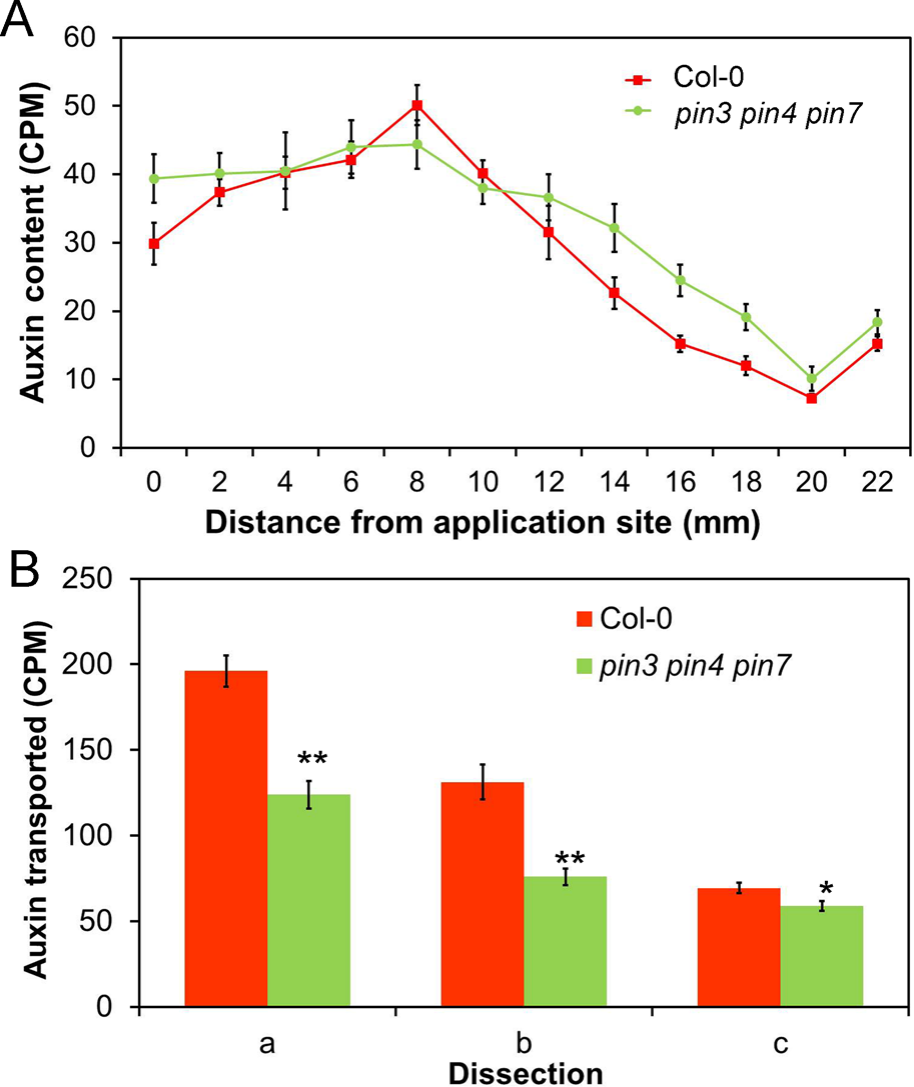
PIN3, PIN4, and PIN7 contribute to auxin exchange between the PATS and surrounding tissues. **A)** Distribution of radio-labelled IAA (measured as CPM) in 2 mm intervals of 24 mm long stem segments 60 min after the immersion of the apical end in 5 μM radio-labelled IAA for 10 min, in Col-0 (red) and *pin3-3 pin4-3 pin7-1* (green). *n* = 8 per time point, bars indicate s.e.m. **B)** Cross-stem transport in Col-0 and *pin3-3 pin4-3 pin7-1*, assayed using the dissections a, b, and c (see Fig 3). *n* = 20, bars indicate s.e.m., asterisks indicate significant differences between genotypes for each treatment; * = *p* < 0.05; ** = *p* < 0.005 (*t* test).

Given their expression and sub-polar localization in the outer xylem parenchyma cells, we reasoned that loss of PIN4 and PIN7 might allow us to probe the role of the apparent low-conductance polar transport regime in auxin movement and shoot branching. We did not detect any reproducible differences in either bulk auxin transport (*t* test, *n* = 16, *p* = 0.46) or pulse shape in the *pin4-3 pin7-1* double mutant relative to wild type (S5 Fig). We therefore analyzed auxin transport in the *pin3-3 pin4-3 pin7-1* triple mutant, since PIN3, PIN4 and PIN7 are very closely related at the protein level and are known to act redundantly in many processes (e.g., [58]). We observed an approximately 25% reduction in bulk auxin transport in *pin3-3 pin4-3 pin7-1* compared to wild type (*t* test, *n* = 20, *p* < 0.0005) (Fig 10B). We also assessed the effect of *pin3-3 pin4-3 pin7-1* on the shape of auxin pulses, and found that, in this background, a proportion of the auxin molecules travels faster than in wild-type stems—but that equally, another proportion travels more slowly than in wild type (Fig 10A).

We used our computational models to understand the basis for these complex contributions of PIN3, PIN4, and PIN7 to stem auxin transport dynamics. The *pin3 pin4 pin7* mutant does not match the predictions of simply reducing the activity of the low conductance polar channel (i.e., the difference between the two-channel and three-channel models, see Fig 6D and 6E). Therefore PIN3, PIN4, and PIN7 are not simply acting as a low-conductance polar channel. Rather, the data suggest that PIN3, PIN4, and PIN7 function both in the PATS and non-PATS cells (consistent with their expression patterns). We could best simulate the *pin3 pin4 pin7* mutant in our model by decreasing the permeability of lateral walls in every channel (S6 Fig), thereby reducing the exchange of auxin between the channels. PIN1 is the major driver of polar transport in an expression domain that overlaps with that of PIN3, PIN4, and PIN7. It is therefore a reasonable hypothesis that loss of PIN3/PIN4/PIN7 would disproportionately affect lateral auxin exchange between the high- and low-conductance channels compared to basal polar transport. Our data suggest that in wild type, there is a high level of exchange between the PATS and adjacent cells (Fig 3). Reduced lateral permeability would result in auxin molecules in the PATS remaining in the PATS, and therefore progressing more consistently and rapidly down the stem. These auxin molecules therefore form a population that moves faster than in wild type. Similarly, reduced lateral permeability would result in auxin molecules in non-PATS cell files being less likely to be transferred into the PATS. These auxin molecules travel more slowly than in wild type and therefore form a population that is left behind at the apical end of the stem. These observations are consistent with PIN3, PIN4, and PIN7 acting both to funnel auxin from the broader stem tissues into the PATS and to disperse auxin from the PATS into the other tissues, and suggest that the lateral component of PIN3, PIN4, and PIN7 localization may be particularly important for this process.

To investigate further the role of these proteins in auxin movement, we assayed cross-stem transport in *pin3-3 pin4-3 pin7-1*. Despite the overall reduction in the amount of radio-label transported through stem segments (Fig 10B, dissection a), the amount transported across the segments was only slightly impaired (*t* test, *n* = 20, *p* = 0.027) (Fig 10B, dissection c). Indeed, as a proportion of the total amount of auxin transported to the basal end of the stem segment, the amount delivered to the opposite side of the stem from the site of application was increased (*t* test, *n* = 20, *p* < 0.0005) (Fig 10B). This result is consistent with the main effect of the triple mutant being to reduce auxin exchange between PATS and non-PATS cells. As a result, auxin in this experiment tends to remain in the cell files into which it is loaded at the apical end of the segment. Only a limited amount of auxin is loaded directly into PATS cell files, which occupy a small proportion of the stem, distributed among the discrete vascular bundles; this auxin moves rapidly and directly down the stem. In contrast, the non-PATS domain occupies a much wider area, which is contiguous across the stem. Reduced exchange between PATS and non-PATS cells in *pin3 pin4 pin7* thus results in a disproportionate reduction in auxin in the PATS, while residual auxin mobility in non-PATS tissues may be supported by ABCB-mediated non-polar transport.

Taken together, our analysis of PIN3, PIN4, and PIN7 supports the notion of an auxin transport activity in the stem that acts in the exchange of auxin between the high-conductance, highly polar transport in the PATS and the non/less-polar regime in other tissues.

### PIN3/PIN4/PIN7 Mediated Auxin Exchange Allows Communication between Tissues

**Fig 11:**
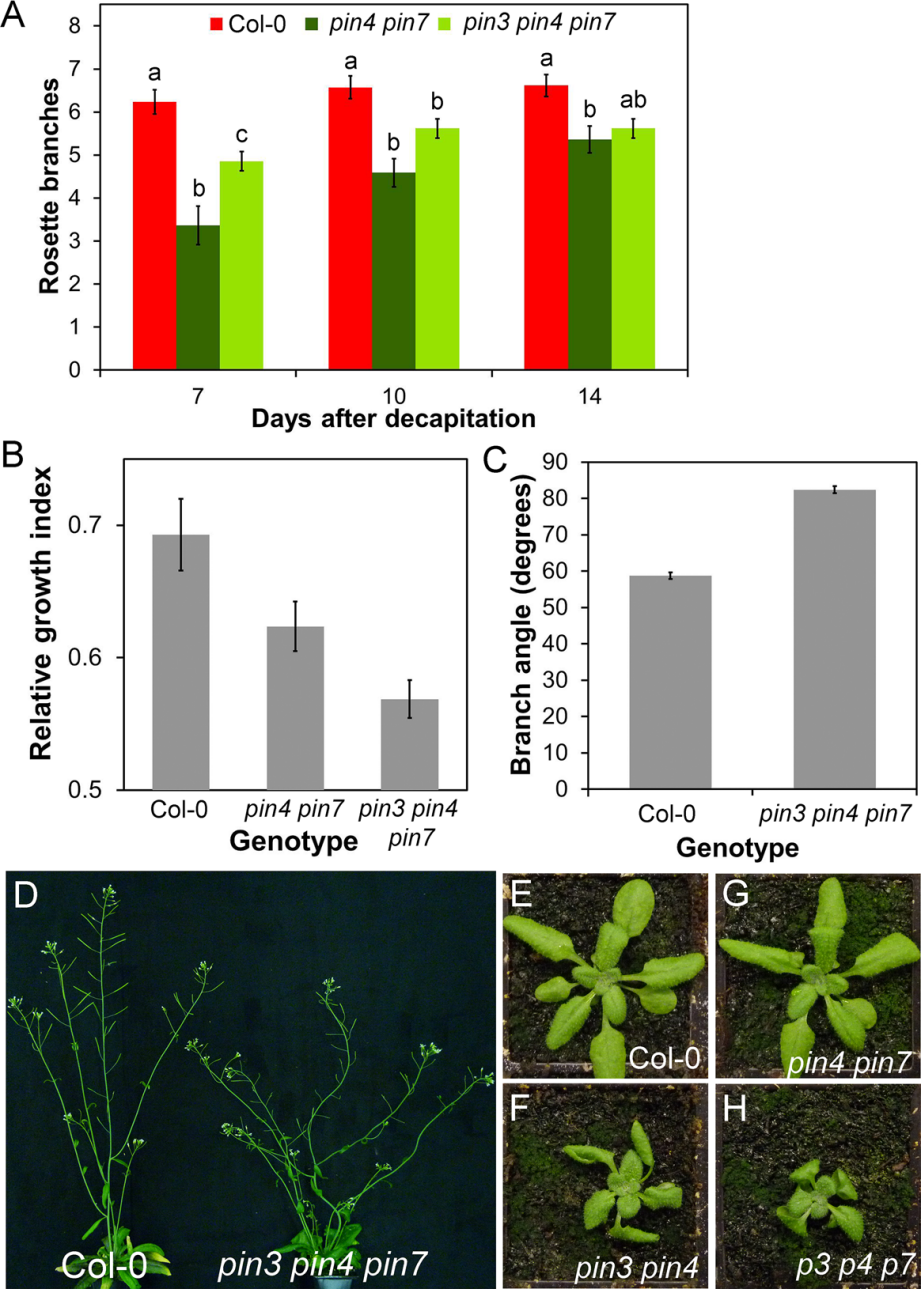
PIN3, PIN4, and PIN7 influence shoot branching. **A)** Rosette branching in Col-0, *pin4-3 pin7-1*, and *pin3-3 pin4-3 pin7-1* after decapitation. *n* = 21–22, bars indicate s.e.m. For each time point, bars with different letters are significantly different from each other (ANOVA, Tukey HSD, *p* < 0.05). **B)** Bud-bud communication in Col-0, *pin4-3 pin7-1*, and *pin3-3 pin4-3 pin7-1*. Explants were decapitated and left for 10 d. The mean relative growth index (longest branch/total branch length) was calculated for each genotype. *n* = 21–24; bars indicate s.e.m. Relative growth index is significantly reduced in *pin4-3 pin7-1* and *pin3-3 pin4-3 pin7-1* relative to Col-0 (ANOVA, *p* < 0.05). **C)** The angle formed between secondary cauline branches and the primary stem at the point of emergence in Col-0 and *pin3-3 pin4-3 pin7-1*. *n* = 45–46 cauline branches from at least 10 plants per genotype; bars indicate s.e.m. The angle is significantly different between the two genotypes (*t* test, *n* = 45–46, *p* < 0.005). **D)** Phenotype of the *pin3-3 pin4-3 pin7-1* shoot system compared to Col-0 (6 wk old). **E–H)** Leaf phenotypes in 4-wk-old rosettes of Col-0 (E), *pin3-3 pin4-3* (F), *pin4-3 pin7-1* (G), and *pin3-3 pin4-3 pin7-1* (H).

To test the functional significance of this PIN3/PIN4/PIN7-mediated auxin exchange activity, we looked for phenotypic effects of *PIN3*, *PIN4*, and *PIN7* mutation on shoot branching, a process which requires communication between shoot apices, both dormant and active, which we have shown is mediated through the auxin transport system [12,34]. We examined the *pin3-3 pin4-3*, *pin4-3 pin7-1*, and *pin3-3 pin4-3 pin7-1* mutants for branching phenotypes. Under our standard long-day growth conditions, we did not observe any consistent alterations in branching, at least in part because wild-type branching is low under these conditions (S7 Fig). We therefore used a more sensitive branching assay in which plants are grown in short days for 4 wk to increase rosette leaf number, transferred to long days to induce flowering and then decapitated [59]. In this assay, we observed reduced shoot branching in all of these mutant combinations compared to wild type, but perhaps more interestingly, the kinetics of bud activation were affected (Fig 11A) (S7 Fig). In Col-0, nearly all post-decapitation bud outgrowth is established in the first 7 d, and while the branches that activate during that time continue to grow longer thereafter, very few additional buds activate (Fig 11A). In contrast, *pin4-3 pin7-1* and *pin3-3 pin4-3 pin7-1* activate a smaller number of branches than wild type in the initial 7 d after decapitation (ANOVA, Tukey HSD, *n* = 21–22, *p* < 0.05), and although this difference reduces over time, they have still activated significantly fewer buds by 14 d (ANOVA, Tukey HSD, *n* = 21–22, *p* < 0.005) (Fig 11A). This late activation is reflected in the shorter mean length of branches in *pin4-3 pin7-1* at 14 d compared to wild type (*t* test, *n* = 12, *p* < 0.005) (S7 Fig).

We have previously shown that the two buds on an excised Arabidopsis stem segment bearing two nodes are able to communicate, and they compete with each other across the stem, such that one bud can inhibit the other [36]. These two buds vascularize into different vascular bundles and, as such, are unlikely to communicate through the PATS or through an acropetally mobile auxin-regulated signal in the xylem such as strigolactone or cytokinin [36]. We hypothesized that this bud-bud communication might be mediated in part through the activity of PIN3, PIN4, and PIN7.

In wild-type two-node stem segments, one branch usually dominates, inhibiting the growth of the other. Either the apical or basal bud can dominate, and the degree of dominance can therefore best be captured using a relative growth index (RGI), defined as the length of the longest branch divided by the summed length of both branches (Fig 11B) [36]. When we analyzed the *pin4-3 pin7-1* and *pin3-3 pin4-3 pin7-1* mutants in this assay, we found that often both buds elongated, giving a reduced relative growth index compared to Col-0 (ANOVA, Tukey HSD, *n* = 21–24, *p* < 0.05) (Fig 11B), suggesting that there is reduced competition between the buds. Our data thus suggest that PIN3, PIN4, and PIN7 are important in mediating lateral communication across the stem.

The redundant functions of PIN3, PIN4, and PIN7 have previously been extensively explored in embryos [39,58], roots [44,58], root gravitropism and gravitropic set point [60–63], and seedling phototropic and photomorphogenic responses [64–67]. However, unlike *pin1*, post-seedling shoot phenotypes for these mutants are not well described. In addition to the branching phenotypes noted above, we also observed apparent defects in the gravitropic set-point angle of branches in *pin3-3 pin4-3*, such that branches emerged from the stem at a more obtuse angle than in wild type (*t* test, *n* = 10, *p* < 0.05) (S7 Fig). This phenotype was enhanced in *pin3-3 pin4-3 pin7-1* (Fig 11C), with stems and branches unable to maintain consistent growth vectors, resulting in twisting and looping (Fig 11D). Furthermore, we observed that leaf development in *pin3-3 pin4-3* and *pin3-3 pin4-3 pin7-1* strongly deviated from wild type, with shorter, twisted blades (Fig 11E, 11F and 11H). We did not observe any major phenotypic defects in *pin4-3 pin7-1* other than in the regulation of shoot branching (Fig 11G). As far as has been described in the literature, PIN3, PIN4, and PIN7 do not play significant roles in organogenesis at the shoot meristem [68] or in vascular patterning in leaves [53], both major transport-driven phenomena. However, the data presented here suggest that PIN3, PIN4, and PIN7-driven auxin transport does play an important role in shaping shoot system architecture in Arabidopsis.

## Discussion

### The Polar Auxin Transport Stream and Auxin Transport Canalization

The Arabidopsis PIN1 protein plays an important role in the PATS, but also in local patterning in the shoot apical meristem and the developing leaf vasculature. The central midvein of newly emerging leaves forms by a canalization-like process (e.g., [49,50]) that connects the midvein with existing vascular bundles in the stem, linking each leaf directly to the PATS through continuous files of PIN1 expressing cells. It is therefore a reasonable hypothesis that the PATS is part of a canalization-dominated transport network, built by dynamic polarization of PIN1 aligned in the direction of auxin flux. This is consistent with the classical experiments of Tsvi Sachs where vascular strands with unusual trajectories can be induced in pea epicotyls (a relatively undifferentiated tissue) simply by manipulating the position and strength of sources of and sinks for auxin [30,31]. However, our data suggest that once established, PIN1 expression patterns and polarities in differentiated Arabidopsis stems are relatively stable, and likely determined by tissue type. There is apparently no requirement for auxin flux to maintain these patterns, and although *PIN1* transcription is regulated by auxin, PIN1 accumulation appears to be limited to particular cell types. Thus the PATS is in effect the endpoint of the canalization process, and once established it is no longer subject to flux-correlated re-modelling, although it could still be subject to dynamic regulation over rapid timescales, for example by changes in PIN1 phosphorylation [49] or strigolactone-induced PIN1 endocytosis [12]. These results are consistent with physiological experiments in various species demonstrating the long term stability of the polarity of the PATS in inverted stem segments [69], consistent with our observations suggesting slow PIN1 cycling in these cells [12], as represented in our previous models [33]. Presumably there are strong functional and structural reasons to sacrifice the dynamism of PIN1 behavior in the stem, for example to maintain the strength of the PATS and the robust arrangement of primary vascular bundles.

### Auxin Is Required for Maintenance of the Polar Auxin Transport Stream

Although auxin cannot repattern the PATS in Arabidopsis stems, it is nevertheless required for the maintenance of the PATS, by promoting localization of PIN1 at the basal plasma membrane. Removal of the shoot apex results in the gradual depletion of PIN1 protein in a basipetal progression along the stem, correlating with a reduction in auxin levels in the stem. Replacement of the apex with applied auxin within ~24 hours prevents or reverses this depletion, but subsequent addition of auxin cannot rapidly restore PIN1 membrane localization. This is consistent with classical data demonstrating the requirement of auxin for maintenance of polar auxin transport (reviewed in [70]). Our data suggest that auxin regulates *PIN1* transcription, along with the endo- and/or exocytosis of the PIN1 protein, resulting in loss of PIN1 in the absence of auxin.

### Connective Auxin Transport and the Polar Auxin Transport Stream

The gradual depletion of auxin that we observe following decapitation suggests that auxin dynamics in the stem are complex, as indeed has been suggested by other recent work [42,71]. While some auxin molecules move rapidly, at a rate of approximately 1.5 cm per hour, other molecules progress an order of magnitude more slowly. Our modelling demonstrates that these results are incompatible with a single mode of auxin transport, but instead can be captured by models in which multiple transport regimes are used, with auxin exchanging between them as it moves down the stem. Minimally, these include a highly polar, high-conductance regime and a non-polar, low-conductance regime, though our results demonstrate that a third, polar low-conductance regime can better reproduce our empirical results. There are alternative explanations for the observed transport kinetics, such as conjugation and then release of the applied radio-labelled auxin as it moves down the stem. However, this seems highly unlikely given our current understanding of auxin conjugation and conjugate hydrolysis in Arabidopsis [72–75]. In contrast, our observations of auxin exporter expression patterns and activities in the stem support our multi-modal transport model.

**Fig 12:**
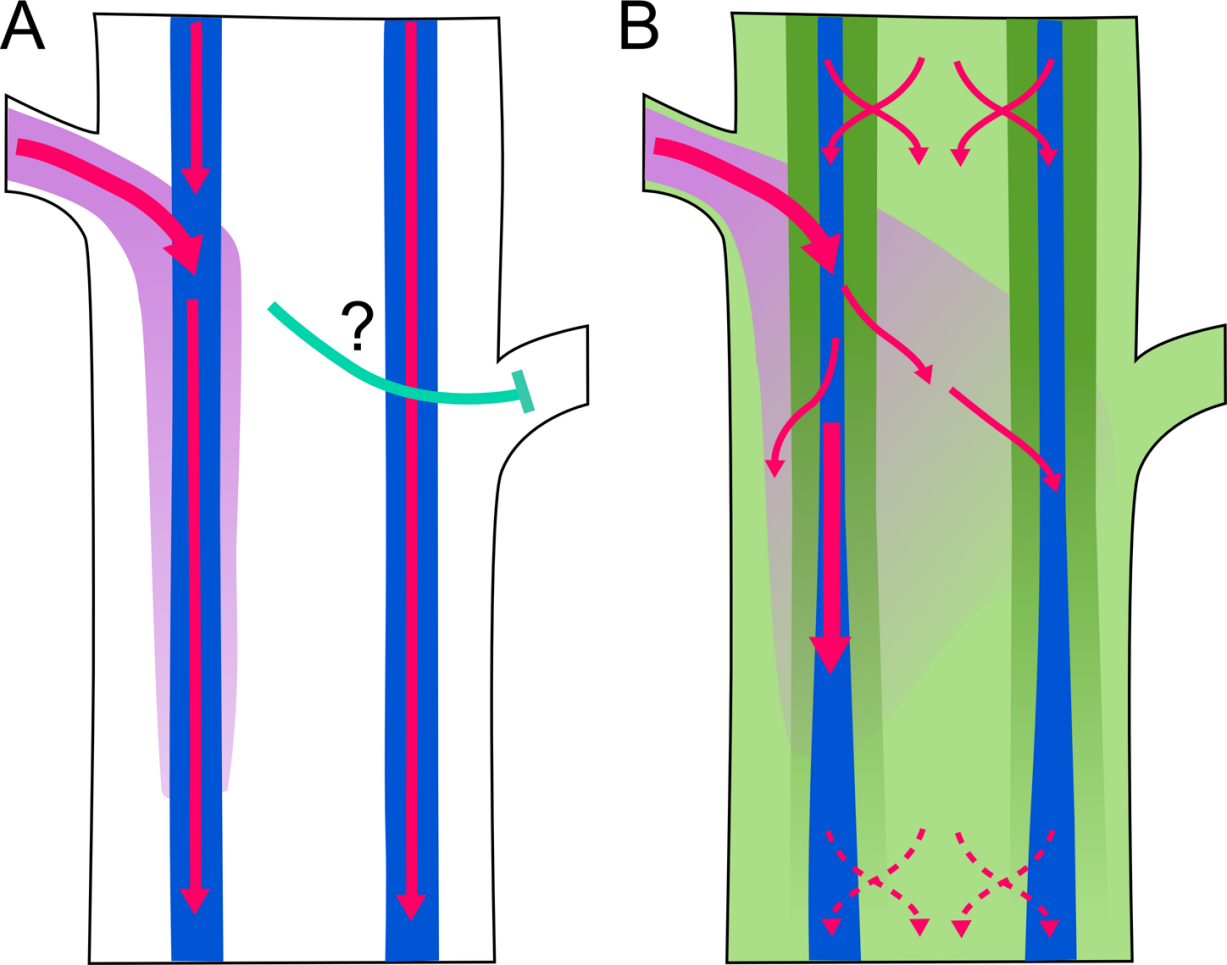
Model of auxin transport-mediated communication in stems. Schematic figure illustrating models of auxin transport in an Arabidopsis stem segment bearing two buds. Pink arrows indicate auxin transport; purple shading indicates concentration of auxin exported from the left-hand bud. Blue shading indicates the position of the high-conductance polar auxin transport stream (PATS), dark green shading indicates low-conductance polar transport, and light green shading indicates low-conductance non-polar auxin transport. The green areas together constitute connective auxin transport (CAT). Auxin is exchanged between PATS and CAT (red crossed arrows). **A)** Auxin transport scheme with only high-conductance polar auxin transport around the vascular bundles. Auxin exported from the bud on the left is rapidly taken up into the PATS on the left. The mechanism of communication between buds (cyan arrow) is unclear. **B)** Auxin transport scheme with widespread auxin transport in the stem. There is widespread exchange of auxin between PATS and CAT tissues, particularly in the younger part of the stem (top). Auxin exported from the bud on the left can move across the stem, providing a mechanism for bud-bud communication. In older parts of the stem (bottom), the decline in PIN4/PIN7 expression reduces auxin exchange between PATS and CAT tissues.

Classical data show that high-conductance auxin transport is associated with the vascular bundles, and particularly the vascular cambium and xylem parenchyma [20,38], constituting the well-documented polar auxin transport stream (PATS). This corresponds to PIN1 expression patterns, and the dramatic effect on auxin transport of PIN1 mutation. We found strong experimental support for non- or less polar, low conductance auxin transport in the stem tissues outside the PATS, which we term “connective auxin transport” (CAT) because of its ability to mediate communication between the PATS and the surrounding tissues and, indeed, between the PATS strands in the stem. Although for pragmatic reasons our models involve two or three distinct auxin transport regimes with exchange between them, the expression patterns of auxin transporters across the stem suggest that there is a gradient of transport activities, with the classical PIN1-dominated PATS at one extreme, ABCB19/1-expressing pith cells at the other, and weakly polar PIN4 and PIN7 in the outer xylem parenchyma in between. The observed age/stage-related changes in PIN7 and PIN4 expression and polarity suggest that as internodes mature, the system transitions from this graded state into a more bimodal system, in which non-polar and highly polar transport activities dominate (Fig 12).

Crucially, our analysis suggests that PIN3, PIN4, and PIN7 play an important role in the lateral exchange of auxin between the PATS and CAT tissues. It is important to emphasize that *PIN3*, *PIN4*, and *PIN7* are expressed broadly in the stem, with a variety of polarities, and are therefore likely to contribute to all auxin transport activities, including the PATS. Similarly, *ABCB1* and *ABCB19* are expressed broadly in the stem and are likely to contribute to all transport regimes, including CAT. These redundancies mean that the activity of PIN3, PIN4, and PIN7 is not equivalent to the low-conductance polar transport regime in our models, but mutation in *PIN3 PIN4* and *PIN7* disproportionately affects auxin exchange between PATS and CAT. Currently available genetic tools did not permit in depth assessment of the role of ABCB1 and ABCB19 in stem auxin transport, and this remains an obvious priority for the future.

### PATS, CAT, and Communication in the Stem

We have recently argued that the properties of the PATS make it an inefficient mechanism if its primary role is simply to move auxin from the shoot to the root, and that a major role for the auxin transport network is in providing sensitive and self-organizing communication between tissues and organs [76]. We thus propose that the PATS functions as an integrated information source, reflecting the combined activity of auxin sources across the shoot, while CAT both distributes this information to the local tissues of the stem, and funnels information from the wider shoot system into the PATS (Fig 12) [76]. In the context of shoot branching control, the auxin level and therefore auxin sink strength in the PATS provides information about the number and health of active apices on the shoot, while CAT provides the interface through which this information is conveyed to each successive bud along the stem. For example, following decapitation, auxin in the PATS may deplete only slowly, in a basipetal progression, because it is replenished by auxin held in the surrounding tissues, which is fed into it by CAT. Axillary buds may thus react to decapitation not because of large changes in the PATS, but because of small changes in auxin level in the peripheral tissues. CAT may thus be particularly important in permitting the initial stages of auxin flux from the bud, which could trigger canalization between bud and stem. Since buds export auxin into the PATS, there may be a seamless transition between apically derived auxin and laterally derived auxin in this scenario, such that auxin levels in the PATS at the node remain relatively stable throughout.

Our analysis of bud activation in the *pin4 pin7* and *pin3 pin4 pin7* mutant combinations is consistent with this idea. The mutants display both negative and positive effects on bud activation. Following decapitation, *pin3 pin4 pin7* triple mutant rosette buds activate more slowly than the wild-type controls, consistent with slower establishment of polar auxin transport between the bud and the PATS in the main stem, because of reduced transport activity in the intermediate tissues. In apparent contrast, on stem segments bearing two cauline nodes, both buds activate more often in the mutants than in the wild-type, where one bud tends to dominate the other. This result is consistent with reduced competition between the branches due to impaired communication, resulting from impaired CAT-mediated auxin exchange between the PATS in the individual vascular bundles of the stem. This interpretation begs the question, which we hope to address in the future, of whether bud-stem canalization acts through PIN1 or PIN3/PIN4/PIN7, or some sequential combination of both activities. The models we have established in the work described here will be an invaluable tool in this endeavor.

## Materials and Methods

### Plant Materials

The *pin1-613*, *PIN1*:*GUS* [10], *pin3-3*, *pin4-3*, *pin7-1*, *pin3-3 pin4-3*, *pin4-3 pin7-1*, *PIN3*:*PIN3-GFP*, *PIN7*:*PIN7-GFP* [54], *PIN1*:*PIN1-GFP* [37], *PIN2*:*PIN2-GFP* [77], *PIN6*:*PIN6-GFP*, *PIN8*:*PIN8-GFP* [53], *ABCB19*:*GFP-ABCB19* [56], and *DR5rev*:*GFP* [39] lines have all been described previously. We created a new PIN4:PIN4-GFP line because stem expression in existing lines was very low and inconsistent (see below). *abcb1-100 (atpgp1-100*, SALK_083649), *abcb1-101* (*atpgp1-101*, SALK_046440), *abcb19-101* (*atmdr1-101*, SALK_033455) and *abcb19-102* (*atmdr1-102* SALK_031406) have been previously described under different aliases [55]. The *abcb1-100 abcb19-101* double mutant was re-isolated in this study by PCR genotyping of candidate F3 lines. Primers used for genotyping mutant combinations are shown in S1 Table.

### Plant Growth Conditions

Plants for analysis were grown on Levington’s F2 compost, under a standard 16 hr/8 hr light/dark cycle (22°C/18°C) in controlled environment rooms with light provided by white fluorescent tubes, (intensity ~150 μMm^-2^s^-1^). Stem segments used for various assays were incubated in the same conditions.

### Cloning

A new *PIN4:PIN4-GFP* reporter line was created by amplifying the genomic sequence of *PIN4* (At2g01420) from -4,598 to +1,032, relative to the start codon (+1), and from +1,033 to +3,095, with SalI linkers. eGFP was amplified with SalI linkers, and ligated between the *PIN4* fragments (i.e., at position +1,032 in *PIN4*) after SalI digest (primers listed in S2 Table). A correctly assembled *PIN4:PIN4-GFP* construct was then transferred into a binary vector for transformation into Arabidopsis, as detailed in [78].

### Hormone Treatments

Plates containing 55 ml of ATS-agar (no sucrose) [79] were made, from which a 1 cm band was removed when set, leaving two 25 ml blocks of agar (after [40]). Hormones could then be added to either the apical or basal block at known concentrations. Naphthalene acetic acid (NAA, Sigma) and 1-*N*-naphthylphthalamic acid (NPA, Sigma) were dissolved in 1 ml DMSO, then diluted to stock concentrations of 1 mM with 50 ml 70% ethanol, then added to plates at the concentrations described. Control plates were treated with a mock 2% DMSO, 70% ethanol solution. Segments 2cm long from the basal internodes (first or second) of primary inflorescence stems were then excised and placed into the split-plates with one end embedded in each block, and left under standard 16/8 hr light/dark growth conditions for the stated durations.

### Bud Competition Assay

Bud-bud competition was determined by taking bolting stems that carried three cauline buds, each less than or equal to 1 mm in length. The shoot apex and the uppermost cauline bud were removed under a dissecting microscope using the tip of a hypodermic needle. The two-node segments were then placed in parafilm-covered eppendorf tubes containing ATS solution [79]. Bud lengths were measured for 10 consecutive days and the relative growth index was determined by calculating the proportion of branch length in the longest branch [36].

### Microscopy

Transverse and longitudinal hand sections were made through basal internodal stem segments and the slices were then embedded in agar plates. A dissecting microscope was used to position the longitudinal sections through vascular bundles. Images were taken using a Zeiss LSM700 imaging system with 20× water immersion lenses. Transmitted light was used to identify the appropriate cell types on the basis of positional and morphological characteristics (using the highly lignified xylem cells as a positional cue). GFP fluorescence was then imaged by laser-scanning confocal microscopy. Excitation was performed using 488 nm (15% laser power) and 555 nm (6%) lasers. Chloroplast autofluorescence was detected above 600 nm, and GFP fluorescence below 555 nm. The same settings for GFP detection were used within experiments for each line, except where stated. GFP quantification was performed on non-saturated images, using Zeiss “ZEN” software. Fluorescence intensity in the GFP channel was measured in five basal plasma membranes per sample, in at least eight independent samples.

### Auxin Quantification

For tissue measurements, 15 mm stem segments were excised from the basal internode of inflorescence stems, and treated as described. Sub-sections of ~10 mg were then purified and analyzed by gas chromatography-tandem mass spectrometry (GC-MS/MS) as described in [80] with minor modifications. To each sample, 500 pg ^13^C_6_-IAA was added as an internal standard before extraction. Four replicates were analyzed for each time point.

For eluate measurements, 15 mm stem segments were excised from the basal internode of inflorescence stems and placed with their basal end in PCR tubes containing 50 μl 2.5 mM sodium diethyldithiocarbamate buffer for a given time; the same segments were then serially transferred to fresh buffer as appropriate during the time-course. The eluate was then purified as described in [33] and analyzed by GC-MS/MS. To each sample, 500 pg ^13^C_6_-IAA was added as an internal standard before extraction. Four replicates were analyzed for each time point.

### Auxin Transport Assays

Standard “bulk” auxin transport assays were modified from those described in [34]. Stem segments 18 mm long from basal internodes were excised, and the apical end submerged in 30 μl ATS without sucrose (pH = 5.6), containing 1 μM ^14^C-IAA (American Radiolabeled Chemicals) in an Eppendorf tube. Stems were incubated for 6 hours, and the basal 5 mm of the segment was then excised, placed in 200 μl scintillation liquid and shaken overnight at 400 RPM prior to scintillation counting. Cross-stem assays were performed in the same manner after appropriate dissection using a scalpel (see Fig 5E). For pulse assays, the apical ends of 24 mm stem segments from basal internodes were submerged for 10 min in 20 μl ATS without sucrose (pH = 5.6), containing 5μM ^14^C-IAA or 250 nM ^3^H-IAA (for *pin1-613* pulses) and 0.005% Triton X-100. Samples were then transferred to fresh ATS solution without radio-label and left for various time periods to allow the radio-label to move through the segments. Samples were then cut into 2 mm segments using stacked razor blades with 2 mm spacers. Each segment was placed in 200 μl scintillation liquid and shaken overnight at 400 RPM prior to scintillation counting.

### qPCR Analysis

For *PIN1*, *MAX4* and *GFP* gene expression analyses (Fig 2A), stem segments were subjected to different auxin treatment regimes as described above and harvested onto liquid nitrogen. Total RNA was extracted using the RNeasy Plant Mini kit (Qiagen, http://www.qiagen.com) and DNAse treated using the Turbo DNA-free kit (Ambion) as per manufacturer’s instructions. RNA was quantified using a NanoDrop 1000. For cDNA synthesis, 500 ng of total RNA was reverse transcribed with Superscript II (Invitrogen, http://www.invitrogen.com) according to manufacturer’s instructions. Quantification of transcript levels was carried out using SYBR Green reactions with 5 ng cDNA in a 20 μL volume on a Light Cycler 480 II (Roche, http://www.roche.com) relative to the reference gene *UBC21* (*UBIQUITIN-CONJUGATING ENZYME 21*; At5g25760). At least two technical replicates were run for each biological replicate and averaged. Expression levels were calculated using the Light Cycler 480 II software and the second derivative maximum method assuming equal primer efficiencies. Primers used are given in S3 Table.

## Supporting Information

S1 Fig Effect of NPA on pulse movement. Distribution of radio-labelled IAA (measured as CPM) in 2 mm intervals of 24 mm long stem segments after application of a 10 min pulse of 5 μM IAA, either in the presence (orange line) or absence (green line) of 10 μM NPA. Stems were dissected and analyzed after 90 min elapsed since the application of the pulse; *n* = 8 per time point, bars indicate s.e.m.
(TIF)

S2 Fig Auxin transport model equations. The pulse assay and bulk transport assay models were implemented in VVE, an extension of VV (Smith 2003). The model physical setup is shown in Fig 6A and the equations used are shown here. Plant cells in the stem vasculature are typically elongated, a feature that was included in the computational model as a 1:10 width:length ratio. For this reason, while the intra-cellular horizontal diffusion timescale was relatively small, the vertical diffusion timescale was more relevant to the computation. The first was therefore neglected, whereas the second was explicitly modeled. No-flux boundary conditions were imposed at the apical edge of the top row of cells and similarly at the basal edge of the bottom row of cells. At the start of the simulations, auxin concentration was set to 0 in all cells, apart from those on the top row, where a fixed concentration was maintained for the duration of the radio-labelled auxin treatment. At the end of a simulation, the bulk assay simulator reported the amount of auxin accumulated in the segment’s basal 5 mm, while the pulse assay simulator reported the amount of auxin accumulated in contiguous 2 mm sub-segments, which we called an auxin profile. Simulated auxin profiles were re-scaled to match the area under the curve of experimental profiles. The auxin drainage simulation model was implemented in a similar way to the other two models, but auxin was allowed to drain from the base of the segment. A background auxin production term *σ* was also added to the auxin time derivative in all cells.
(TIF)

S3 Fig Reduced permeability in high- and low-conductance channels recapitulates auxin transport in *pin1*. **A):** Three-channel model simulation of pulse assay, manually fitted to wild-type pulse profile (black line) by using the following parameter values (mm/min): high conductance polar channel: p_1_ = 2, q_1_ = 0.2; q_12_ = 9 10^−4^; low conductance polar channel: p_2_ = 0.3, q_2_ = 0.7, q_21_ = q_22_ = q_23_ = 0.01; non-polar channel: q_3_ = 0.3, q_32_ = 2.5 10^−4^. **B–D):** Attempts to simulate measured pulse profiles from the *pin1-613* mutant (black line) by manually altering p1 and p2 parameter values. A 4-fold (B) or 8-fold reduction (C) in p1 relative to (A) fails to capture the behavior of *pin1*. However, a 4-fold reduction in both p1 and p2 relative to (A) recapitulates the behavior of *pin1* (D). All other parameters were as in (A). **E)** Bulk auxin transport in *pin1-613* as a percentage of wild type after 6 or 18 hours incubation. For the measured data, error bars show the s.e.m, *n* = 20–23. Simulations of bulk transport assays using reduced permeability in the high- and low-conductance polar channels more closely match the measured data than reducing permeability in the high conductance channel alone. Parameter values are as in pulse simulations B–D.
(TIF)

S4 Fig *PIN4* expression is dynamic during stem development. **A,B)** *PIN1*:*PIN1-GFP* expression in the cambium and xylem parenchyma, transverse (A) and longitudinal sections (B) of basal internode of 30 cm tall (= 6/7 wk old) inflorescence stem. These images also appear in Fig 8. **C,D)** *PIN4*:*PIN4-GFP* expression in transverse (C) and longitudinal sections (D) of basal internodes of 3 cm tall (= 5 wk old) inflorescence stems. **E–H)** *PIN4*:*PIN4-GFP* expression in transverse (E,G) and longitudinal sections (F,H) of basal (E,F) and apical (G,H) internodes of 10 cm tall (= 5/6 wk old) inflorescence stems. Part of image G is shown in Fig 7C.
(TIF)

S5 Fig Auxin transport dynamics in *pin4 pin7*. **A**) Bulk basipetal auxin transport (measured in CPM) in the basal internodes of 6 wk old Col-0 and *pin4-3 pin7-1* plants. *n* = 16, bars indicate s.e.m. There is no significant different between the two genotypes (*t* test, *p* = 0.46). **B)** Distribution of radio-labelled IAA (measured as CPM) in 2 mm intervals of 24 mm long stem segments 60 min after application of a 10 min apical pulse of 5μM IAA, in Col-0 (blue) and *pin4-3 pin7-1* (red). *n* = 8, bars indicate s.e.m.
(TIF)

S6 Fig Reduced lateral permeability recapitulates auxin transport in *pin3 pin4 pin7*. Simulations of auxin pulse profiles in wild-type **(A)** and *pin3 pin4 pin7* (**B**), compared to measured profiles (*n* = 8, error bars show the s.e.m). **A)** Parameter values (mm/min): high conductance polar channel: p_1_ = 4, q_1_ = 0.2; q_12_ = 0.9 10^−3^; low conductance polar channel: p_2_ = 0.3, q_2_ = 0.7, q_21_ = q_22_ = q_23_ = 10^−2^; non-polar channel: q_3_ = 0.3, q_32_ = 0.6 10^−3^; **B)** As in (A), apart from: q_12_ = 0.45 10^−3^ and q_21_ = q_22_ = q_23_ = 5 10^−3^.
(TIF)

S7 Fig Shoot phenotypes in *pin3 pin4* and *pin4 pin7*. **A)** Rosette branching in long-day grown Col-0, *pin3-3 pin4-3*, *pin4-3 pin7-1*. *n* = 10–12, bars indicate s.e.m. For each time point, bars with the same letter are not significantly different from each other (ANOVA, *p* < 0.05). **B)** Rosette branching in short-day/long-day decapitation assay, measured 7 or 14 d after decapitation in Col-0, *pin3-3 pin4-3*, *pin4-3 pin7-1*. *n* = 11–12, bars indicate s.e.m. Bars with the same letter are not significantly different from each other (*t* test, *p* < 0.05). **C)** Average length of branches formed in (B) 14 d after decapitation in Col-0, *pin3-3 pin4-3*, *pin4-3 pin7-1*. *n* = 58–80 branches, bars indicate s.e.m. Bars with the same letter are not significantly different from each other (*t* test, *p* < 0.05). **D)** The angle formed between secondary cauline branches and the primary stem at the point of emergence in Col-0 and *pin3-3 pin4-3*. *n* = 37 branches from 10 plants for each genotype. The mean was calculated per plant, then averaged across the 10 plants; bars indicate standard error of this mean. The angle is significantly different between the two genotypes (*t* test, *n* = 10, *p* < 0.005).branches, bars indicate s.e.m.
(TIF)

S1 Table Mutant genotyping. Genotyping strategies for PIN3, PIN4, and PIN7 are modified from [58].
(DOCX)

S2 Table Primers for cloning. (DOCX)

S3 Table Primers for qPCR analysis. (DOCX)

S1 Text Automated parameter fitting. (RTF)

## Abbreviations

ABCB: ATP-binding cassette B family
CAT: Connective Auxin Transport
CPM: Counts per minute
GC-MS: Gas chromatography-mass spectrometry
GFP: Green fluorescent protein
IAA: Indole-3-acetic acid
NAA: Naphthalene acetic acid
NPA: 1-*N*-naphthylphthalamic acid
PATS: Polar Auxin Transport Stream
PIN: PIN FORMED
RGI: relative growth index
SAMs: shoot apical meristems
