# Supplementary figures and images for: Connective Auxin Transport in the Shoot Facilitates Communication between Shoot Apices
Source: PLoS Biol. 2016 Apr 27;14(4):e1002446. doi: 10.1371/journal.pbio.1002446 (PMC4847802; doi:10.1371/journal.pbio.1002446)

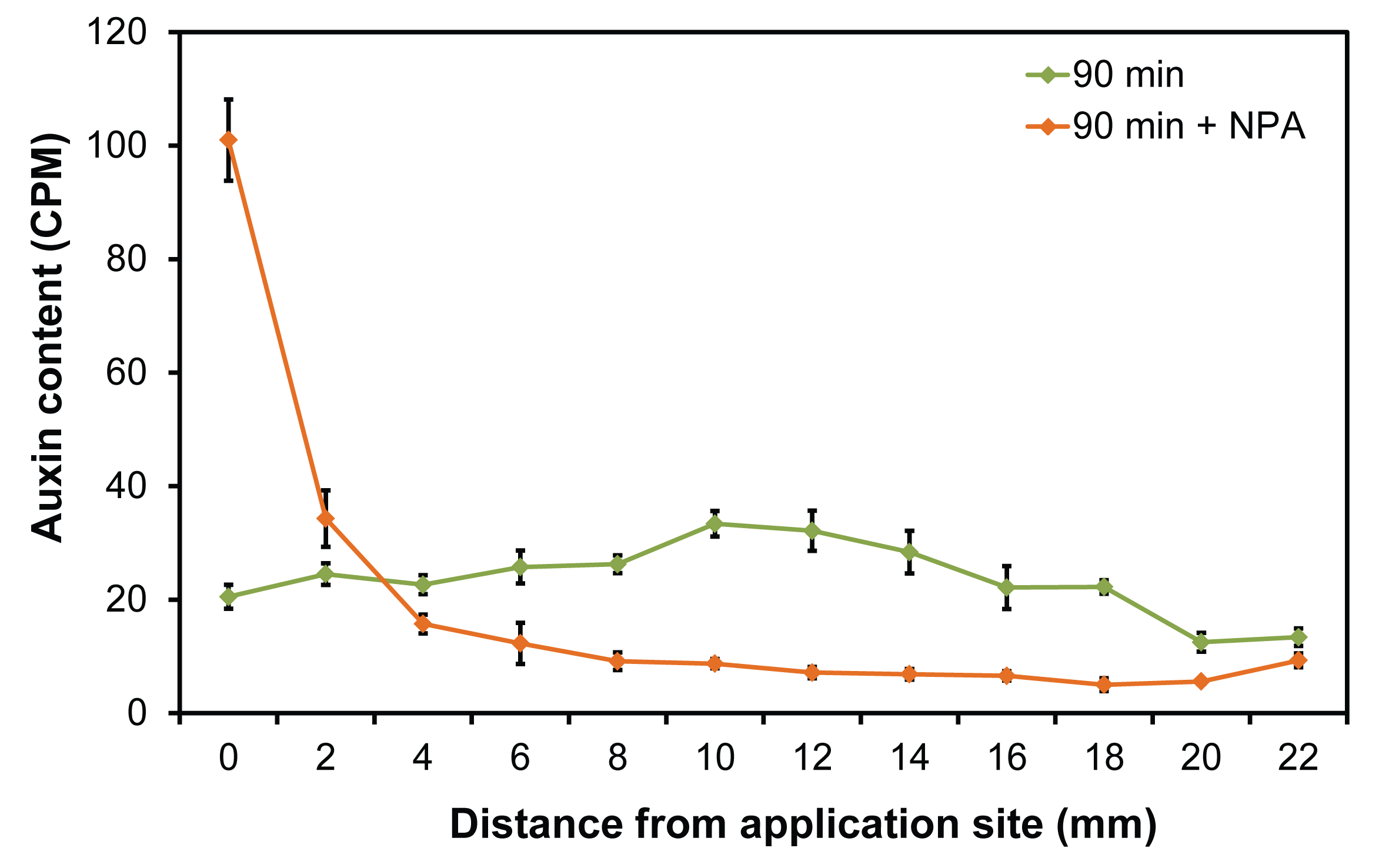

Supplement: S1 Fig — Distribution of radio-labelled IAA (measured as CPM) in 2 mm intervals of 24 mm long stem segments after application of a 10 min pulse of 5 μM IAA, either in the presence (orange line) or absence (green line) of 10 μM NPA. Stems were dissected and analyzed after 90 min elapsed since the application of the pulse; n = 8 per time point, bars indicate s.e.m. (TIF) [file pbio.1002446.s001.tif]

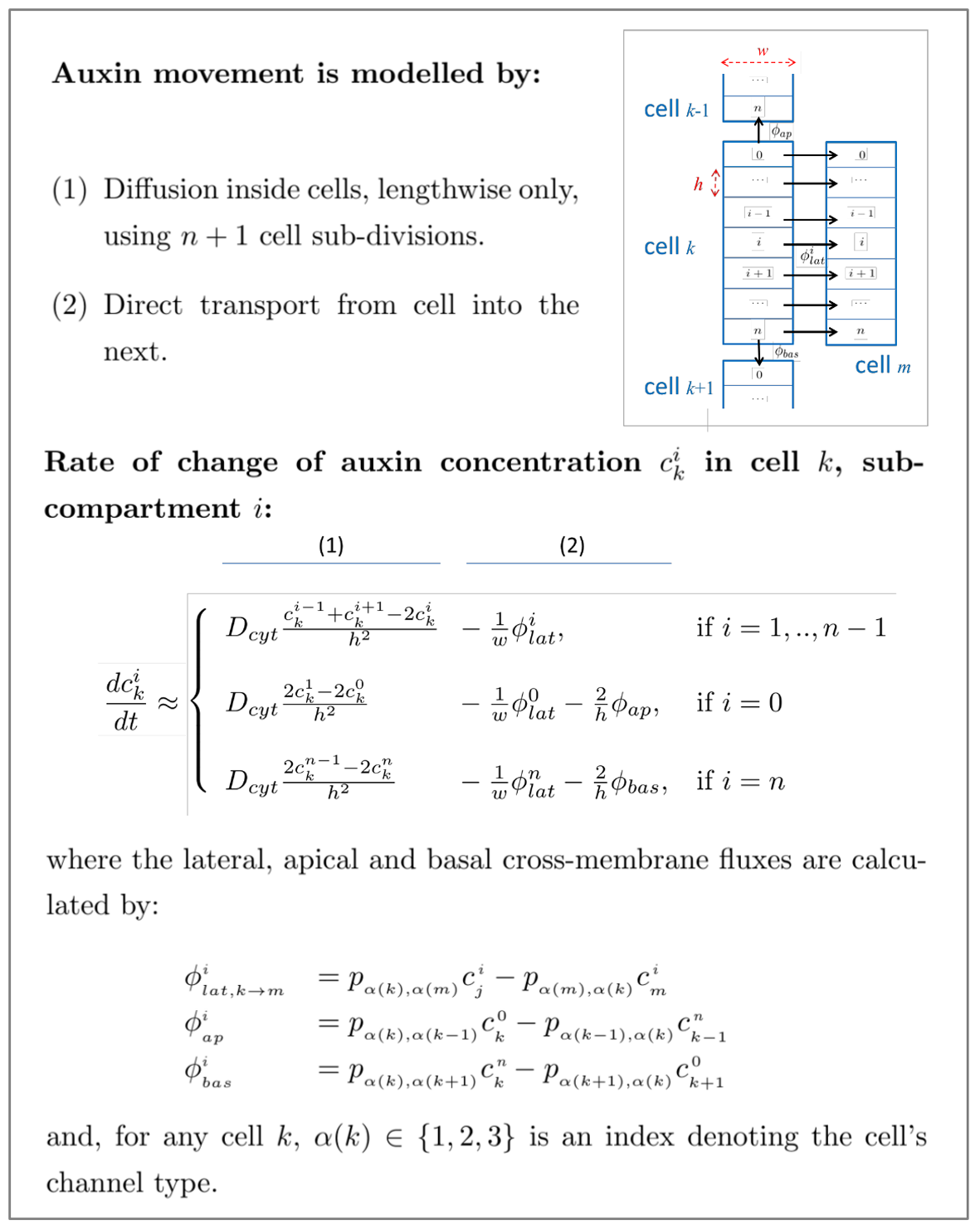

Supplement: S2 Fig — The pulse assay and bulk transport assay models were implemented in VVE, an extension of VV (Smith 2003). The model physical setup is shown in Fig 6A and the equations used are shown here. Plant cells in the stem vasculature are typically elongated, a feature that was included in the computational model as a 1:10 width:length ratio. For this reason, while the intra-cellular horizontal diffusion timescale was relatively small, the vertical diffusion timescale was more relevant to the computation. The first was therefore neglected, whereas the second was explicitly modeled. No-flux boundary conditions were imposed at the apical edge of the top row of cells and similarly at the basal edge of the bottom row of cells. At the start of the simulations, auxin concentration was set to 0 in all cells, apart from those on the top row, where a fixed concentration was maintained for the duration of the radio-labelled auxin treatment. At the end of a simulation, the bulk assay simulator reported the amount of auxin accumulated in the segment’s basal 5 mm, while the pulse assay simulator reported the amount of auxin accumulated in contiguous 2 mm sub-segments, which we called an auxin profile. Simulated auxin profiles were re-scaled to match the area under the curve of experimental profiles. The auxin drainage simulation model was implemented in a similar way to the other two models, but auxin was allowed to drain from the base of the segment. A background auxin production term σ was also added to the auxin time derivative in all cells. (TIF) [file pbio.1002446.s002.tif]

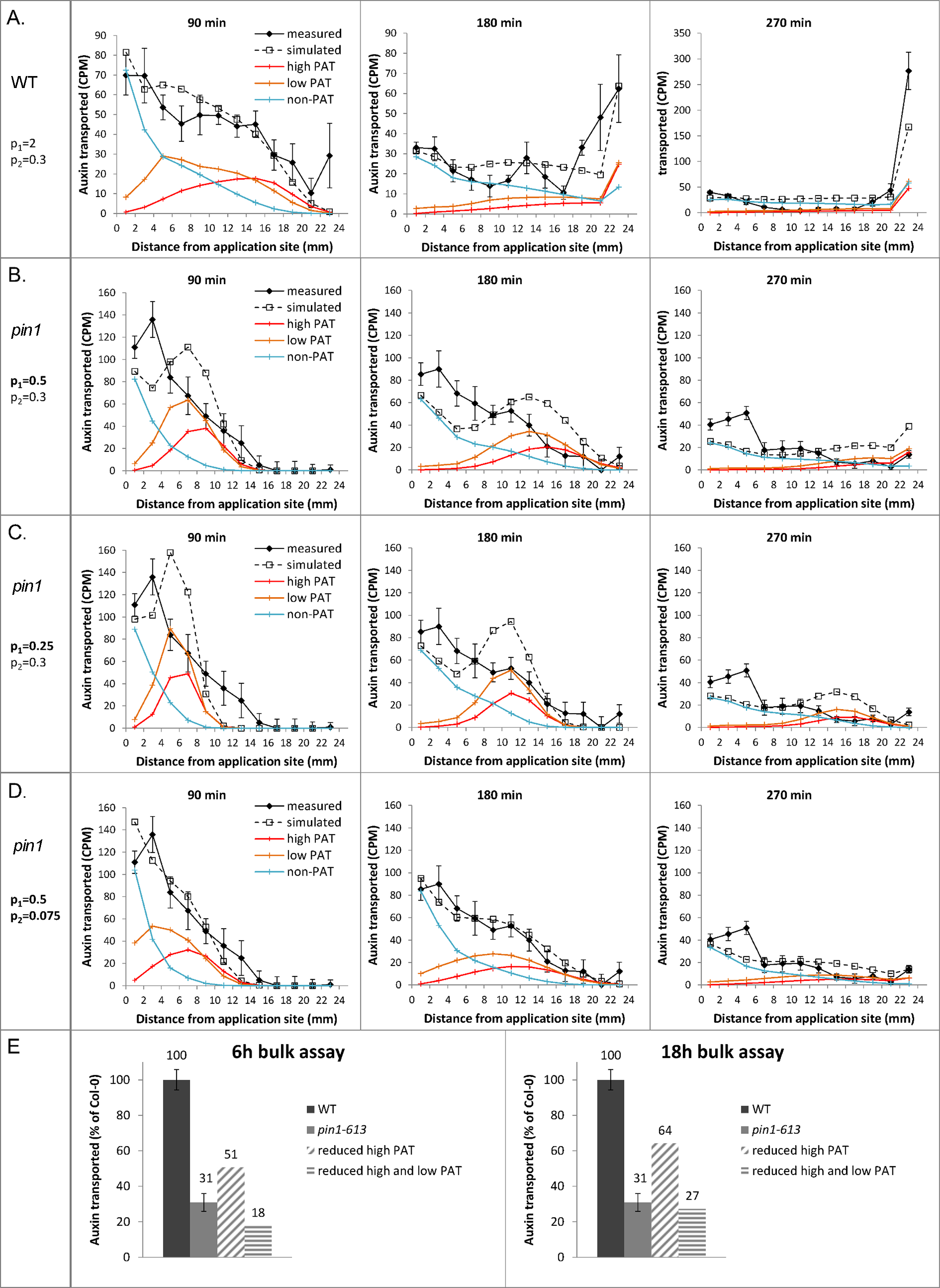

Supplement: S3 Fig — A): Three-channel model simulation of pulse assay, manually fitted to wild-type pulse profile (black line) by using the following parameter values (mm/min): high conductance polar channel: p1 = 2, q1 = 0.2; q12 = 9 10−4; low conductance polar channel: p2 = 0.3, q2 = 0.7, q21 = q22 = q23 = 0.01; non-polar channel: q3 = 0.3, q32 = 2.5 10−4. B–D): Attempts to simulate measured pulse profiles from the pin1-613 mutant (black line) by manually altering p1 and p2 parameter values. A 4-fold (B) or 8-fold reduction (C) in p1 relative to (A) fails to capture the behavior of pin1. However, a 4-fold reduction in both p1 and p2 relative to (A) recapitulates the behavior of pin1 (D). All other parameters were as in (A). E) Bulk auxin transport in pin1-613 as a percentage of wild type after 6 or 18 hours incubation. For the measured data, error bars show the s.e.m, n = 20–23. Simulations of bulk transport assays using reduced permeability in the high- and low-conductance polar channels more closely match the measured data than reducing permeability in the high conductance channel alone. Parameter values are as in pulse simulations B–D. (TIF) [file pbio.1002446.s003.tif]

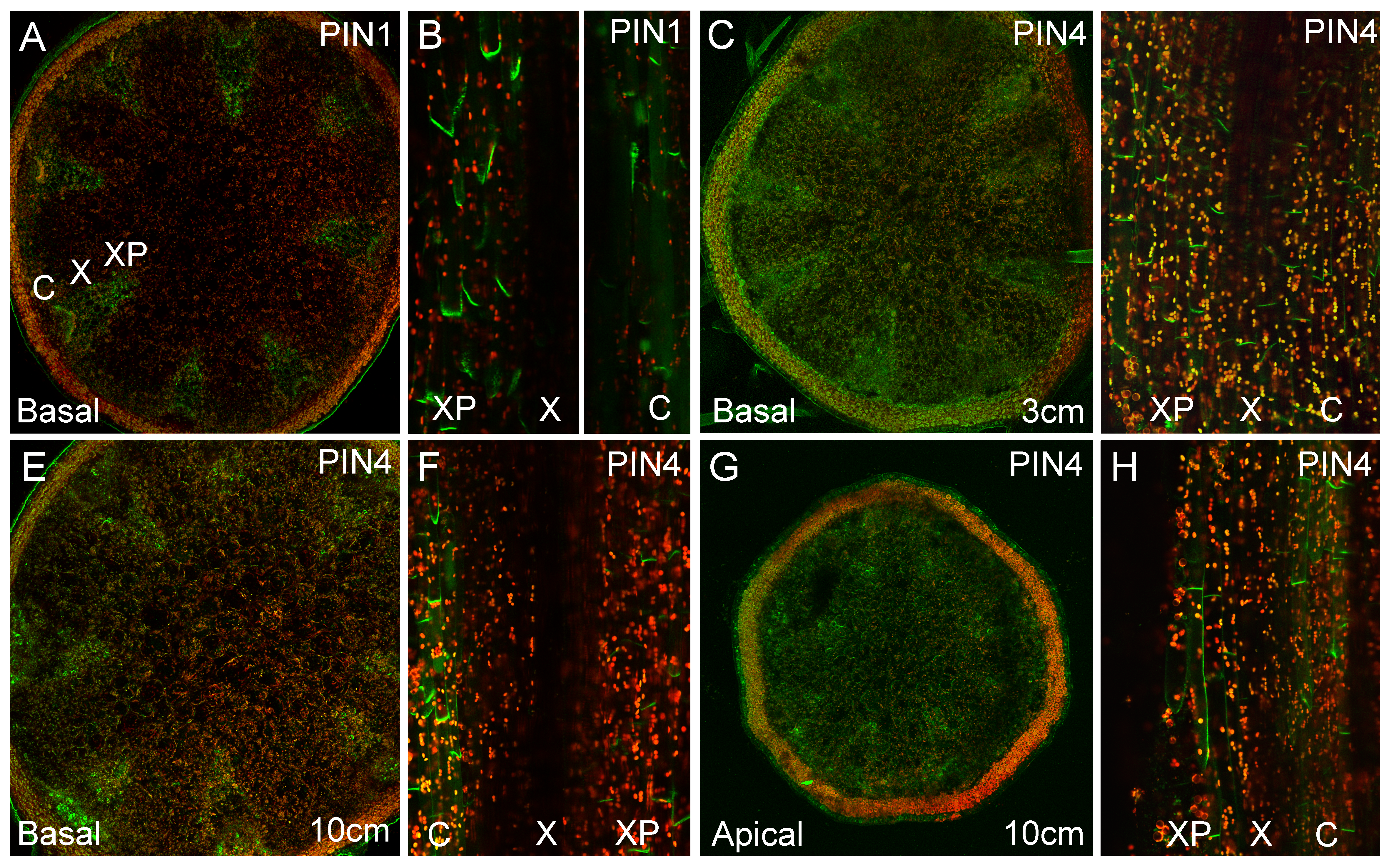

Supplement: S4 Fig — A,B) PIN1:PIN1-GFP expression in the cambium and xylem parenchyma, transverse (A) and longitudinal sections (B) of basal internode of 30 cm tall (= 6/7 wk old) inflorescence stem. These images also appear in Fig 8. C,D) PIN4:PIN4-GFP expression in transverse (C) and longitudinal sections (D) of basal internodes of 3 cm tall (= 5 wk old) inflorescence stems. E–H) PIN4:PIN4-GFP expression in transverse (E,G) and longitudinal sections (F,H) of basal (E,F) and apical (G,H) internodes of 10 cm tall (= 5/6 wk old) inflorescence stems. Part of image G is shown in Fig 7C. (TIF) [file pbio.1002446.s004.tif]

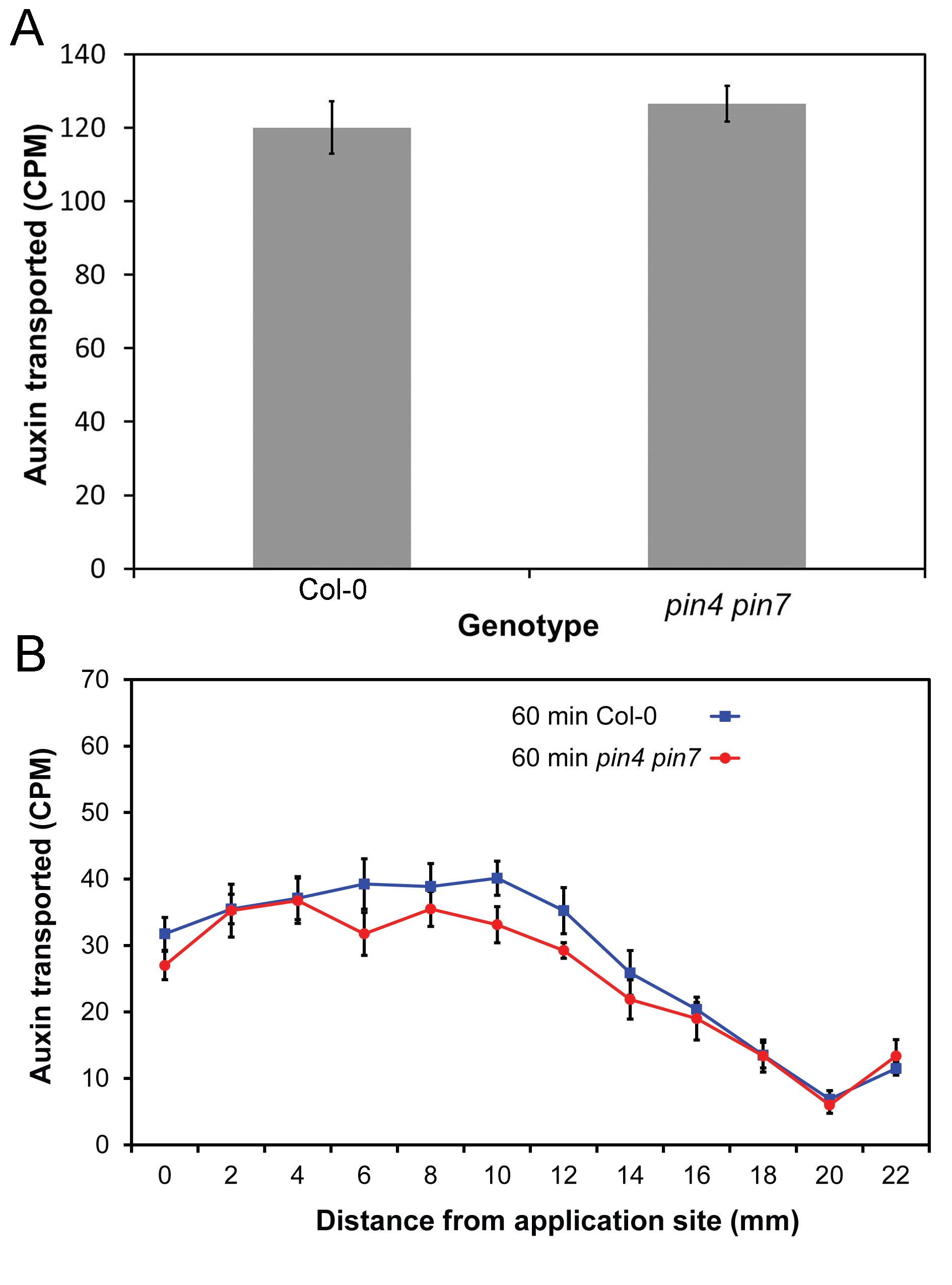

Supplement: S5 Fig — A) Bulk basipetal auxin transport (measured in CPM) in the basal internodes of 6 wk old Col-0 and pin4-3 pin7-1 plants. n = 16, bars indicate s.e.m. There is no significant different between the two genotypes (t test, p = 0.46). B) Distribution of radio-labelled IAA (measured as CPM) in 2 mm intervals of 24 mm long stem segments 60 min after application of a 10 min apical pulse of 5μM IAA, in Col-0 (blue) and pin4-3 pin7-1 (red). n = 8, bars indicate s.e.m. (TIF) [file pbio.1002446.s005.tif]

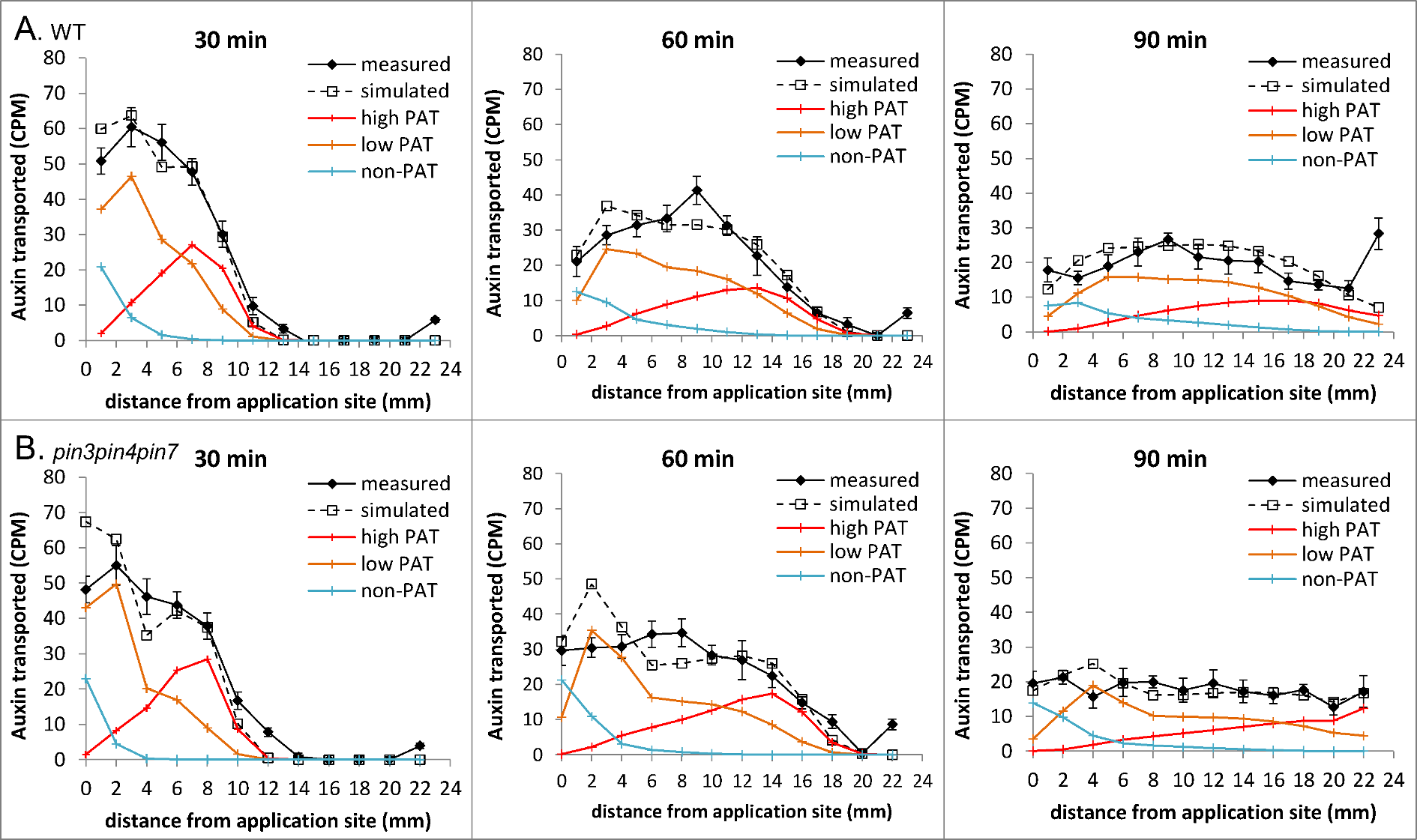

Supplement: S6 Fig — Simulations of auxin pulse profiles in wild-type (A) and pin3 pin4 pin7 (B), compared to measured profiles (n = 8, error bars show the s.e.m). A) Parameter values (mm/min): high conductance polar channel: p1 = 4, q1 = 0.2; q12 = 0.9 10−3; low conductance polar channel: p2 = 0.3, q2 = 0.7, q21 = q22 = q23 = 10−2; non-polar channel: q3 = 0.3, q32 = 0.6 10−3; B) As in (A), apart from: q12 = 0.45 10−3 and q21 = q22 = q23 = 5 10−3. (TIF) [file pbio.1002446.s006.tif]

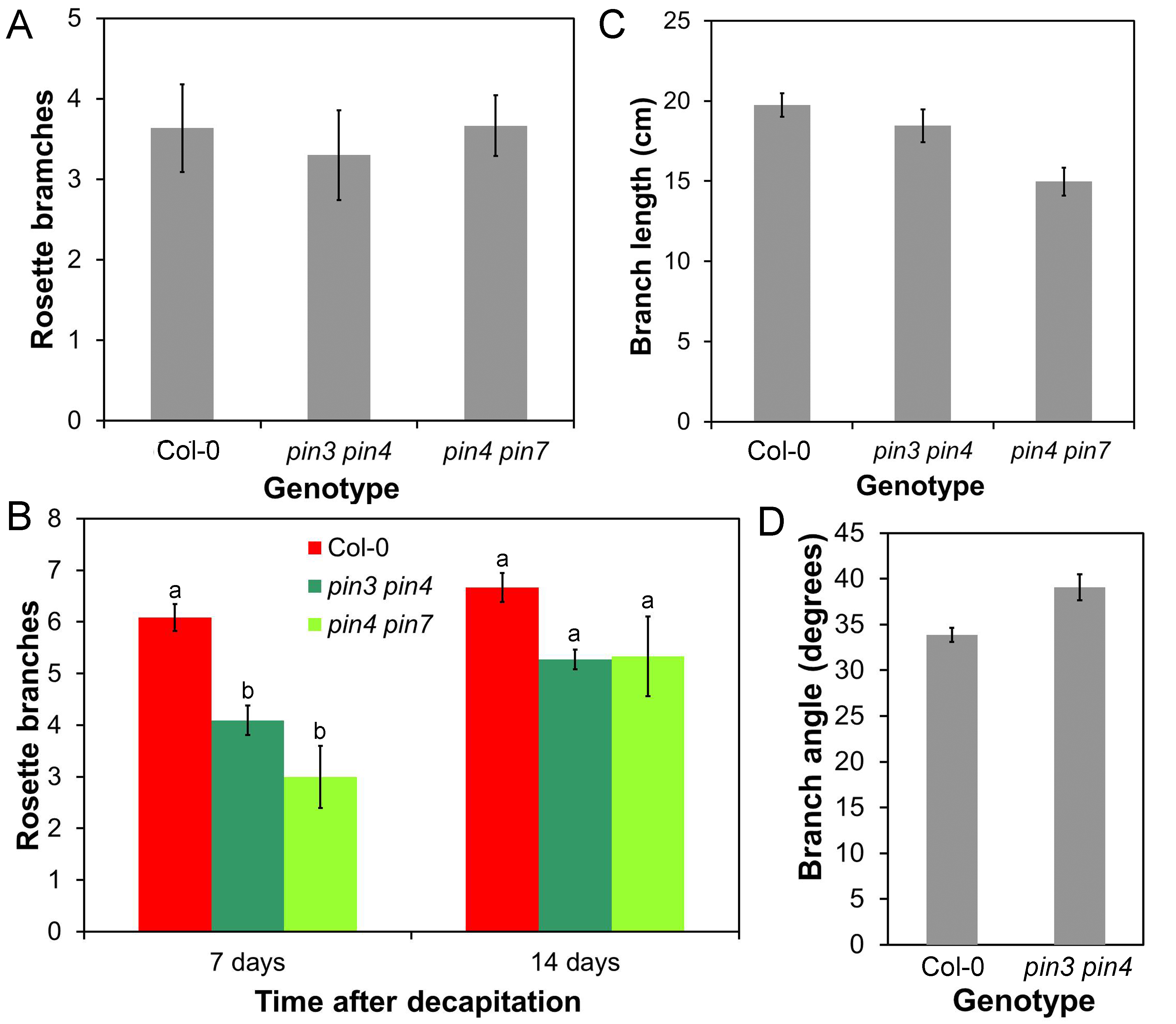

Supplement: S7 Fig — A) Rosette branching in long-day grown Col-0, pin3-3 pin4-3, pin4-3 pin7-1. n = 10–12, bars indicate s.e.m. For each time point, bars with the same letter are not significantly different from each other (ANOVA, p < 0.05). B) Rosette branching in short-day/long-day decapitation assay, measured 7 or 14 d after decapitation in Col-0, pin3-3 pin4-3, pin4-3 pin7-1. n = 11–12, bars indicate s.e.m. Bars with the same letter are not significantly different from each other (t test, p < 0.05). C) Average length of branches formed in (B) 14 d after decapitation in Col-0, pin3-3 pin4-3, pin4-3 pin7-1. n = 58–80 branches, bars indicate s.e.m. Bars with the same letter are not significantly different from each other (t test, p < 0.05). D) The angle formed between secondary cauline branches and the primary stem at the point of emergence in Col-0 and pin3-3 pin4-3. n = 37 branches from 10 plants for each genotype. The mean was calculated per plant, then averaged across the 10 plants; bars indicate standard error of this mean. The angle is significantly different between the two genotypes (t test, n = 10, p < 0.005).branches, bars indicate s.e.m. (TIF) [file pbio.1002446.s007.tif]
