# Supplementary material for: Connective Auxin Transport in the Shoot Facilitates Communication between Shoot Apices
Source: PLoS Biol. 2016 Apr 27;14(4):e1002446. doi: 10.1371/journal.pbio.1002446 (PMC4847802; doi:10.1371/journal.pbio.1002446)
Supplement: S1 Table — Genotyping strategies for PIN3, PIN4, and PIN7 are modified from [58]. (DOCX) [file pbio.1002446.s008.docx]

**Table S9: Mutant genotyping**

Genotyping for PIN3, PIN4 and PIN7 is modified from [58].

| **Name** | **Sequence (5’ to 3’)** |
| --- | --- |
| pin1-613 LP | CAAAAACACCCCCAAAATTTC |
| pin1-613 RP | AATCATCACAGCCACTGATCC |
| LBb1.3 | ATTTTGCCGATTTCGGAAC |
| pin3-3 LP* | GGAGCTCAAACGGGTCACC |
| pin3-3 RP | TAACCGGAAAGCGACGAGA |
| pin4-3 LP | CCTAAAGAAAACCAACAGCA |
| pin4-3 RP | AATTAAACACACAGACCCAC |
| pin7-1 LP | TCCTCGTCCGTCTAATCT |
| pin7-1 RP | CCACATCCCACCTTCATATC |
| En8130 | GAGCGTCGGTCCCCACACTTCTATAC |
| SALK_046440 LP | CTCCGAGTTAAATGCAGCAAC |
| SALK_046440 RP | GAAAAGCTGACCTTGTTGCTG |
| SALK_083649 LP | GAAGACTGCGACAAGGACAAG |
| SALK_083649 RP | GCAAGAGCGATGTTGAAGAAC |
| SALK_033455 LP | GCAATTGCAATTCTCTGCTTC |
| SALK_033455 RP | CTCAGGCAATTGCTCAAGTTC |
| SALK_031406 LP | AGATTCTCTCGGCTATAGCGG |
| SALK_031406 RP | CTATGGAAAGCCTGATGCAAC |

Genotyping strategies

| *pin1-613* | pin1-613 LP + pin1-613 RP; pin1-613 RP + LBb1.3 |
| --- | --- |
| *pin3-3* | pin3-3 LP + pin3-3 RP. Digest of 535 bp fragment with StyI. StyI restriction site is absent in *pin3-3*. |
| *pin4-3* | pin4-3 LP + pin4-3 RP; pin4-3 RP + En8130 |
| *pin7-1* | pin7-1 LP + pin7-1 RP; pin7-1 RP + En8130 |
| *abcb1-101* | SALK_046440 LP + SALK_046440 RP; SALK_046440 RP + LBb1.3 |
| *abcb1-100* | SALK_083649 LP + SALK_083649 RP; SALK_083649 RP + LBb1.3 |
| *abcb19-101* | SALK_033455 LP + SALK_033455 RP; SALK_033455 RP + LBb1.3 |
| *abcb19-102* | SALK_031406 LP + SALK_031406 RP; SALK_031406 RP + LBb1.3 |
