# Supplementary material for: Connective Auxin Transport in the Shoot Facilitates Communication between Shoot Apices
Source: PLoS Biol. 2016 Apr 27;14(4):e1002446. doi: 10.1371/journal.pbio.1002446 (PMC4847802; doi:10.1371/journal.pbio.1002446)
Supplement: S2 Table — (DOCX) [file pbio.1002446.s009.docx]

**Table S10: Primers for cloning**

| PIN4 prom PstI forw | TCTCTGCAGTTTGTGTATCTTAATTATTTGAGTATG |
| --- | --- |
| PIN4 1032 SalI rev | TATGTCGACGTCATGGCTCGCTTTGCTATC |
| PIN4 1033 SalI forw | TATGTCGACGCTAAGGAGCTTCACATG |
| PIN4 UTR EcoRI rev | TACGAATTCCAGTATAAACCACTTAACTAGAAAC |
| EGFP SalI Forw | TATGTCGACGTGAGCAAGGGCGAGGAG |
| EGFP SalI Rev | TATGTCGACCTTGTACAGCTCGTCCATGC |
