# Supplementary material for: Connective Auxin Transport in the Shoot Facilitates Communication between Shoot Apices
Source: PLoS Biol. 2016 Apr 27;14(4):e1002446. doi: 10.1371/journal.pbio.1002446 (PMC4847802; doi:10.1371/journal.pbio.1002446)
Supplement: S3 Table — (DOCX) [file pbio.1002446.s010.docx]

**Table S11: Primers for qPCR analysis**

| PIN1 F | GAAACGCTCCGGTGGTTG |
| --- | --- |
| PIN1 R | GACCAGGTGATGCCGAATA |
| MAX4 F | GAGAAGTCGTGGCTAGCGTG |
| MAX4 R | TGATGACCGTCGCTTTTCC |
| GFP F | tggagttgtcccaattcttgt |
| GFP R | tcaccctctccactgacaga |
| UBC21 F | TCCTCTTAACTGCGACTCAGG |
| UBC21 R | GCGAGGCGTGTATACATTTG |
