# Supplementary material for: Connective Auxin Transport in the Shoot Facilitates Communication between Shoot Apices
Source: PLoS Biol. 2016 Apr 27;14(4):e1002446. doi: 10.1371/journal.pbio.1002446 (PMC4847802; doi:10.1371/journal.pbio.1002446)
Supplement: S1 Text — (RTF) [file pbio.1002446.s011.rtf]

Parameter fitting
Two methods were used to fit the 2-channel and 3-channel model parameters to the WT 30min, 60 min and 90 min pulse data displayed on Fig 5.  

Manual fitting was first used, the process providing an intuitive understanding of the role of the different parameters in the pulse shapes. In particular, it became apparent that lateral permeabilities between channels had to be small by comparison to vertical permeabilities between cells in a cell file. If lateral and vertical permeabilities were of the same order of magnitude, different channels acted as one and the spreading of the pulse as it moves down the stem was limited. As shown on Fig 5, good fits were obtained by this method; incidentally the 3-channel model was better fitted to the data than the 2-channel model. 

Automatic parameter fitting was then performed for the two models, in order to explore the parameter space more widely. We used the Nelder-Mead simplex algorithm	https://www.gnu.org/software/gsl/manual/html_node/Multimin-Algorithms-without-Derivatives.html#Multimin-Algorithms-without-Derivatives (GNU Scientific Library). Parameter scaling was used to search for lateral permeability values on an order of magnitude smaller than vertical permeabilities (When fitting the 2-channel model: parameter scaling for p1 was 1; for q1, q2, scaling was 10; for q21, q22, scaling was 103; for q12, scaling was 104. When fitting the 3-channel model: parameter scaling for p1 was 1; for  p2, q1, q2, q3 , scaling was 10; for q21, q22, q23, scaling was 103; for q12, q32, scaling was 104). The algorithm was run repeatedly for each model, the initial parameters were selected at random each time. (The random number generator simulated the uniform distribution over the following intervals (mm/min): a) when fitting the 2-channel model: p1 was in (0,4); q1, q2 were in (0,1); q12, q21, q22 were in (0,10-3); b) when fitting the 3-channel model: p1 was in (0,4); p2, q1, q2, q3  were in (0,1); q12, q21, q22, q23, q32 were in (0,10-3).) 

Parameter exploration
The 3-channel model was fitted 13 times to the data. The total error (sum of the point-wise absolute errors between simulated and measured profiles at all three time points) was less than 11 for ten solutions, and around 50 for the remaining three. 
The three poor solutions were characterised by much smaller vertical permeabilities in the high conductance channel (p1, q1), and sometimes in the low conductance channel too (p2, q2). The following paragraphs describe parameter sets leading to good solutions only.

Parameter value variations across solutions were relatively small for lateral permeability parameters (fold-change from minimum to maximum between 2 and 4). Fast and slow polar parameters varied slightly more (fold-changes: 4 (p1), 11 (p2)), and apolar vertical parameters varied more again (fold-changes: 40 (q1), 17 (q2), 6 (q3)).

The ratio of polar to non-polar permeabilities in the high conductance (p1/q1, min=5.3, mean=8.5, max=13.9) and low conductance (p2/q2, min=0.65, mean=0.76, max=1.03) channels was generally conserved in these solutions (linear correlation coefficients: corr(p1,q1) >.99 and corr(p2,q2) >.99). 

Lateral permeability coefficients at the interface between the low conductance and apolar channels were highly correlated (corr(q23,q32)> 0.94) and so were the lateral permeabilities at the interface between the slow and high conductance channels and within the slow conductance channel (corr(q21,q12) >0.92, corr(q21,q22) < -0.86). This indicated that a slower transport within the slow conductance channel tended to be compensated by a higher exchange rate with the high conductance channel.

A total of 10831 sets of parameters were trialled by the simplex algorithm whilst producing these 13 solutions.

The 2-channel model was fitted 18 times to the data. The total error was constantly higher than for the 3-channel solutions (between 36 and 91) . Polar (p1) and non-polar (q1) permeabilities in the high conductance channel were again highly correlated in these solutions (corr(p1,q1) >.93; p1/q1, 1st quantile=14.6, median=22.7, 3rd quantile=36.3). The simplex algorithm sampled the parameter space 7147 times in total.
